# A thermally processed cafeteria diet reproducibly mimics early metabolic syndrome through obesity, insulin resistance, and inflammatory activation in Wistar rats

**DOI:** 10.1042/BSR20260388

**Published:** 2026-10-23

**Authors:** Susana Barbosa Ribeiro, Rômulo Camilo de Oliveira Melo, Lucas Vinicius Souza de Carvalho, Arnóbio Antônio da Silva Júnior, Raimundo Fernandes de Araújo Júnior, Amanda Silveira da Silva, Gabrielle Marques de Paiva, Vinicus Barreto Garcia, Ana Heloneida de Araújo Morais, Ana Júlia Felipe Camelo Aguiar, Caroline Addison Carvalho Xavier de Medeiros, Maiara de Morais, Bruna Rafaela Martins dos Santos, Rafael Lino Mokwa, Francisco Caninde de Souza Junior, Maíra Conceição Jerônimo de Souza Lima, Ciro Dantas Soares, Aurigena Antunes de Araújo

**Affiliations:** 1 Postgraduate Program in Pharmaceutical Science, Department of Pharmaceutical Science, Federal University of Rio Grande Norte, Natal, Brazil; 2 Postgraduate Program in Oral Science, Department of Dentistry, Federal University of Rio Grande Norte, Natal, Brazil; 3 Department of Pharmaceutical Science, Federal University of Rio Grande Norte, Natal, Brazil; 4 Postgraduate Program in Pharmaceutical Science, Postgraduate Program in Health Sciences, Department of Pharmaceutical Science, Federal University of Rio Grande Norte, Natal, Brazil; 5 Postgraduate Program in Health Sciences, Postgraduate Program in Functional and Structural Biology UFRN, Department of Morphology, Federal University of Rio Grande Norte, Natal, Brazil; 6 Department of Pharmaceutical Science, Federal University of Rio Grande Norte, Natal, Brazil; 7 Postgraduate Program in Pharmaceutical Science, Department of Pharmaceutical Science, Federal University of Rio Grande Norte, Natal, Brazil; 8 Faculty of Health Sciences (FCS), Federal University of Rondonópolis (UFR), Rondonópolis, Brazil; 9 Postgraduate Program in Biochemistry and Biology, Postgraduate Program in Nutrition, Department of Nutrition, Federal University of Rio Grande Norte, Natal, Brazil; 10 Postgraduate Program in Biochemistry and Molecular Biology, Department of Biochemistry, Federal University of Rio Grande Norte, Natal, Brazil; 11 Department of Biophysical and Pharmacology, Federal University of Rio Grande Norte, Natal, Brazil; 12 Departament of Patology, Federal University of Rio Grande Norte, Natal, Brazil; 13 Graduate Program in Health Science and Technology, Department of Dentistry, State University of Paraíba, Campina Grande, Paraíba, Brazil; 14 Department of Pharmaceutical Science, Federal University of Rio Grande Norte, Natal, Brazil; 15 Postgraduate Program in Pharmaceutical Science, Department of Pharmaceutical Science, Federal University of Rio Grande Norte, Natal, Brazil; 16 Department of Biophysical and Pharmacology, Federal University of Rio Grande Norte, Anclivepa Natal Faculty, Natal, Rio Grande do Norte, Brazil; 17 Graduate Program in Oral Pathology, Faculty of Dentistry of Piracicaba, University of Campinas (UNICAMP), Piracicaba, São Paulo, Brazil; Postgraduate Program in Oral Sciences, Department of Dentistry, Federal University of Rio Grande do Norte, Natal, Rio Grande do Norte, Brazil; 18 Postgraduate Program in Oral Sciences, Postgraduate Program in Pharmaceutical Sciences Department of Biophysical and Pharmacology, Federal University of Rio Grande Norte, Natal, Brazil

**Keywords:** Early metabolic syndrome, Insulin resistance, Thermally processed cafeteria diet

## Abstract

The obesogenic nutrient composition and processing characteristics of ultra-processed foods may contribute to metabolic dysfunction. The present study evaluated a standardized cafeteria diet, alone or combined with low-dose streptozotocin (STZ), in male Wistar rats. Animals (*n* = 32) were allocated to Healthy Control, STZ (35 mg/kg), Cafeteria Diet (CAF), and CAF + STZ groups. The diet contained standard chow, condensed milk, refined sugar, hydrogenated fat, soybean oil, and chocolate-filled biscuits, homogenized and thermally processed at 180°C. After 102 days, anthropometric, metabolic, inflammatory, gene expression, microbiota, and histopathological parameters were assessed. CAF induced a marked obesogenic phenotype, with increased body weight (+172.3%) and abdominal circumference (+38.7%; *P* < 0.01). CAF showed hyperglycemia (median glucose, 403 mg/dl) and higher HOMA-IR than Healthy Control (*P* < 0.01); CAF + STZ also showed increased HOMA-IR (*P* < 0.05). CAF increased IL-1β and IL-6 (*P* < 0.05), triglycerides, adipocyte hypertrophy (*P* < 0.01), and adipose leptin expression (*P* < 0.05), while reducing muscle Slc2a4/GLUT4 expression (*P* < 0.01). Liver, kidney, and pancreatic histopathological scores did not differ significantly. Gut microbiota showed marked compositional imbalance and low taxonomic evenness, with Firmicutes (99.83%), Ligilactobacillus (99.36%), and Lactobacillaceae (99.52%) predominating, while Enterobacteriaceae represented 0.14%. Despite no reduction in Shannon or Simpson indices, extreme Ligilactobacillus dominance indicated a restricted and uneven community. Overall, the cafeteria diet reproduced obesity-associated metabolic dysfunction, characterized by hyperglycemia, insulin resistance, inflammation, adipocyte hypertrophy, altered glucose and leptin expression, and substantial microbiota restructuring.

## Introduction

Metabolic syndrome is a multifactorial metabolic disorder characterized by the coexistence of central obesity, dyslipidemia, hypertension, and impaired glucose metabolism, conditions that collectively increase the risk of cardiovascular disease and type 2 diabetes mellitus. The syndrome is strongly associated with insulin resistance and chronic low-grade inflammation, representing a major public health concern worldwide due to its growing prevalence and its role as a precursor to several cardiometabolic diseases [1].

The development of metabolic syndrome has been closely linked to modern lifestyle factors, particularly dietary patterns characterized by high consumption of saturated fats, refined carbohydrates, and ultra-processed foods. These dietary habits promote excessive caloric intake, adipose tissue expansion, low-grade systemic inflammation, and progressive impairment of insulin signaling pathways. Such metabolic disturbances contribute to the development of obesity and insulin resistance, which are considered central mechanisms underlying the pathophysiology of metabolic syndrome [2].

Experimental animal models based on dietary manipulation have been widely used to reproduce the metabolic alterations observed in humans with metabolic syndrome [3]. Among these approaches, the cafeteria diet has gained considerable attention due to its high palatability, energy density, and ability to induce hyperphagia and rapid weight gain [3,4]. Unlike purified high-fat diets, the cafeteria diet more closely resembles the human Western dietary pattern because it consists of a combination of ultra-processed foods rich in fats, sugars, and salt [5,6]. As a result, this model effectively reproduces several key metabolic disturbances, including obesity, dyslipidemia, insulin resistance, and systemic inflammatory responses [3,7].

Experimental animal models based on dietary manipulation have been widely used to reproduce metabolic alterations associated with metabolic syndrome [3]. Among these approaches, the cafeteria diet has gained considerable attention because of its high palatability, energy density, and capacity to promote hyperphagia and rapid weight gain [8,9]. Compared with purified high-fat diets, cafeteria diets more closely reproduce important features of Western dietary patterns by providing a variety of highly palatable, energy-dense foods, frequently rich in fats, sugars, and salt [9,10]. Consequently, cafeteria feeding can induce several metabolic features relevant to human metabolic syndrome, including obesity, dyslipidemia, insulin resistance, and low-grade inflammation [3,8].

Previous studies have demonstrated that prolonged exposure to cafeteria diets can induce significant metabolic alterations in rodents, particularly increased adiposity, hyperinsulinemia, and reduced insulin sensitivity. While this combined approach effectively produces stable hyperglycemia, it introduces a cytotoxic component that does not fully reflect the natural progression of metabolic disorders in humans, which typically evolve from insulin resistance associated with metabolic syndrome rather than from direct β-cell destruction [5,8,11].

Studies in male Wistar rats have shown that a cafeteria diet alone can induce progressive metabolic disturbances, including increased body weight, visceral adiposity, dyslipidemia, and insulin resistance after prolonged exposure. These metabolic alterations closely resemble the pathophysiological features of metabolic syndrome and represent an early stage in the progression toward type 2 diabetes. Therefore, experimental models based exclusively on cafeteria diet feeding may be particularly useful for investigating the mechanisms underlying diet-induced metabolic dysfunction and insulin resistance [4,8].

Traditional free-choice cafeteria diet models are highly effective in inducing obesity and metabolic dysfunction; however, they frequently introduce substantial inter-animal variability due to differences in food selection and preference, which may affect experimental reproducibility and mechanistic interpretation [9]. In addition, these models often overlook a biologically relevant aspect of the modern Western diet: the chemical transformations generated during food processing, particularly those arising from high-temperature preparation [12,13].

Thermal processing of carbohydrate- and lipid-rich food matrices promotes the formation of Maillard reaction products, advanced glycation precursors, and flavor-active compounds, which not only increase palatability but may also act as direct metabolic stressors capable of modulating inflammation, insulin signaling, and gut microbial ecology [12]. Thus, the biological impact of ultra-processed diets likely depends not only on nutrient composition, but also on the structural, sensory, and chemical properties generated during preparation [14,15].

Therefore, the aim of the present study was to establish a reproducible rat model of early metabolic syndrome using a standardized, thermally processed cafeteria diet and to characterize the metabolic, inflammatory, molecular, histopathological, and gut microbiota alterations associated with the early development of obesity, insulin resistance, impaired glucose homeostasis, and metabolic dysfunction. We hypothesized that chronic exposure to a standardized, thermally processed cafeteria diet would reproducibly induce an early metabolic syndrome-like phenotype characterized by increased adiposity, insulin resistance, low-grade inflammation, impaired glucose homeostasis, adipose tissue remodeling, alterations in insulin-related gene expression, and gut microbiota dysbiosis in Wistar rats.

## Materials and methods

### Experimental design and animals

Male Wistar rats (*Rattus norvegicus*), aged 6 weeks (42 days) and weighing 180–220 g at baseline, were obtained from the Vivarium of the Department of Biophysics and Pharmacology, Federal University of Rio Grande do Norte (UFRN), Natal, RN, Brazil. The animal facility is authorized by the National Council for the Control of Animal Experimentation (CONCEA)/Ministry of Science, Technology and Innovation (MCTI), Brazil, for breeding and maintenance of laboratory animals used in experimental research.

Animals were maintained under standardized environmental conditions (22 ± 2°C, 12 h light/dark cycle) with unrestricted access to standard chow and water. All animal experiments were carried out at the Pharmacology Laboratory, Department of Biophysics and Pharmacology, UFRN, Natal, RN, Brazil. Experimental procedures were performed in accordance with institutional ethical regulations and national guidelines for animal experimentation and were approved by the Institutional Animal Ethics Committee (CEUA/UFRN; protocol no. 044/2025).

### Control and experimental groups

A total of 34 animals were initially included in the experimental protocol and allocated to four groups: healthy control (*n* = 6), streptozotocin (STZ) (*n* = 7), cafeteria diet (*n* = 7), and cafeteria diet combined with low-dose STZ (*n* = 14). During the induction of the experimental model, two animals in the STZ group died, resulting in five animals remaining in this group for the subsequent experimental procedures. No animal losses occurred in the healthy control, cafeteria diet, or cafeteria diet plus low-dose STZ groups.

The larger number of animals initially allocated to the cafeteria diet plus low-dose STZ group was intentionally established considering the variability inherent to the induction of metabolic disease models and the possibility of experimental losses associated with STZ administration. This approach was adopted to ensure an adequate number of animals would remain available for the planned experimental analyses. In addition, the combination of a cafeteria diet with low-dose STZ is a widely used experimental approach for reproducing metabolic alterations resembling metabolic syndrome and type 2 diabetes mellitus, combining diet-induced insulin resistance with partial impairment of pancreatic β-cell function.

- Negative Control (Health): animals fed a standard commercial diet throughout the experiment, without STZ administration (*n* = 6).
- STZ group (STZ): animals fed a standard diet and receiving STZ (35 mg/kg, i.p.) (*n* = 5).
- Cafeteria Diet + STZ (Cafeteria + STZ): animals fed a cafeteria diet for 42 days followed by STZ administration, maintaining the cafeteria diet thereafter (*n* = 14).
- Cafeteria Diet (Cafeteria): animals fed a cafeteria diet for the entire experimental period, without STZ (*n* = 7).

### Standard chow

Nuvilab CR-1 Irradiated (laboratory animal feed; Quimtia, Brazil) was used as the standard diet for nutritional maintenance of the rats. According to the manufacturer’s specifications, the diet is a nutritionally balanced formulation containing plant-derived ingredients, vegetable oil, mineral salts, amino acids, vitamins, trace minerals, and antioxidant additives. The bromatological composition of the diet, as reported by the manufacturer, was as follows:

- Crude protein (minimum): 220 g/kg (22%)
- Ether extract/lipids (minimum): 50 g/kg (5%)
- Crude fiber (maximum): 70 g/kg (7%)
- Mineral matter/ash (maximum): 90 g/kg (9%)
- Moisture (maximum): 125 g/kg (12.5%)
- Calcium: 10–14 g/kg
- Phosphorus (minimum): 6000 mg/kg

The standard chow provided an energy density of approximately 3.39 kcal/g (339 kcal/100 g). The feed was irradiated to ensure microbiological control and enhance sanitary safety for use in experimental animal facilities. Animals had free access to the standard chow and water throughout the experimental period.

### Cafeteria diet composition and preparation

The cafeteria diet was formulated to provide high caloric density and consisted of a mixture of standard chow (Nuvilab CR-1 Irradiated), condensed milk, refined sugar, hydrogenated vegetable fat, soybean oil, and crushed chocolate-filled cookies. This composition resulted in a hyperpalatable diet rich in simple sugars, saturated and trans fatty acids, and omega-6 fatty acids, with low fiber content, promoting hyperphagia, dyslipidemia, and insulin resistance.

As shown in Table 1, the nutritional composition of the cafeteria diet reflects a potent obesogenic profile, characterized by a high energy density (427.3 kcal/100 g). The formulation presents substantial carbohydrate content (59.1 g), high lipid levels (18.5 g), and a relatively low protein concentration (8.9 g), with markedly reduced dietary fiber (2.1 g). The high proportion of refined carbohydrates, derived from the synergy between sucrose (crystal sugar and ultra-processed filled cookies) and lactose (condensed milk), contributes to a high glycemic load. Concurrently, the lipid fraction—sourced from margarine, soybean oil, and ultra-processed biscuit fats—provides a pro-inflammatory profile rich in interesterified, saturated, and omega-6 fatty acids, ideal for inducing metabolic dysregulation.

**Table 1:** Nutritional composition and estimated caloric density of the standardized thermally processed cafeteria diet used to induce early metabolic syndrome in male Wistar rats

Nutricional	Quantitation
Energy value	427.3 kcal
Carbohydrates	59.1 g
Protein	8.9 g
Total fat	18.5 g
Dietary fiber	2.1 g

The diet consisted of the following components: 300 g of crushed ultra-processed filled cookies, 250 g of ground standard chow, 250 g of condensed milk, 100 g of refined sugar, 90 g of margarine, and 10 g of soybean oil (Table 2). The filled biscuit used in the experimental diet presented the following nutritional composition per serving (30 g, corresponding to approximately three cookies): approximately 140 kcal (588 kJ), 21 g carbohydrates, including 12 g sugars, 5.9 g total fat (1.8 g saturated fat), 1.5 g protein, 0.9 g dietary fiber, and 98 mg sodium.

**Table 2:** Ingredient composition and proportional formulation of the standardized thermally processed cafeteria diet administered to male Wistar rats during experimental induction of metabolic syndrome

Ingredients	Total prodution (g)	Cafeteria diet (g) per 100 g
Chow (standard feed)	250	25
Condensed milk	250	25
Cristal sugar	100	10
Margarine	90	9
Soybean oil	10	1
Ultra-processed filled cookies	300	30
Total	1000	100

Initially, all solid components (biscuits and standard chow) were finely ground and homogenized. Separately, condensed milk, margarine, and soybean oil were mixed until a uniform semi-solid mass was obtained. This mixture was then incorporated into the dry ingredients and thoroughly homogenized to ensure consistency (Figure 1).

**Figure 1:**
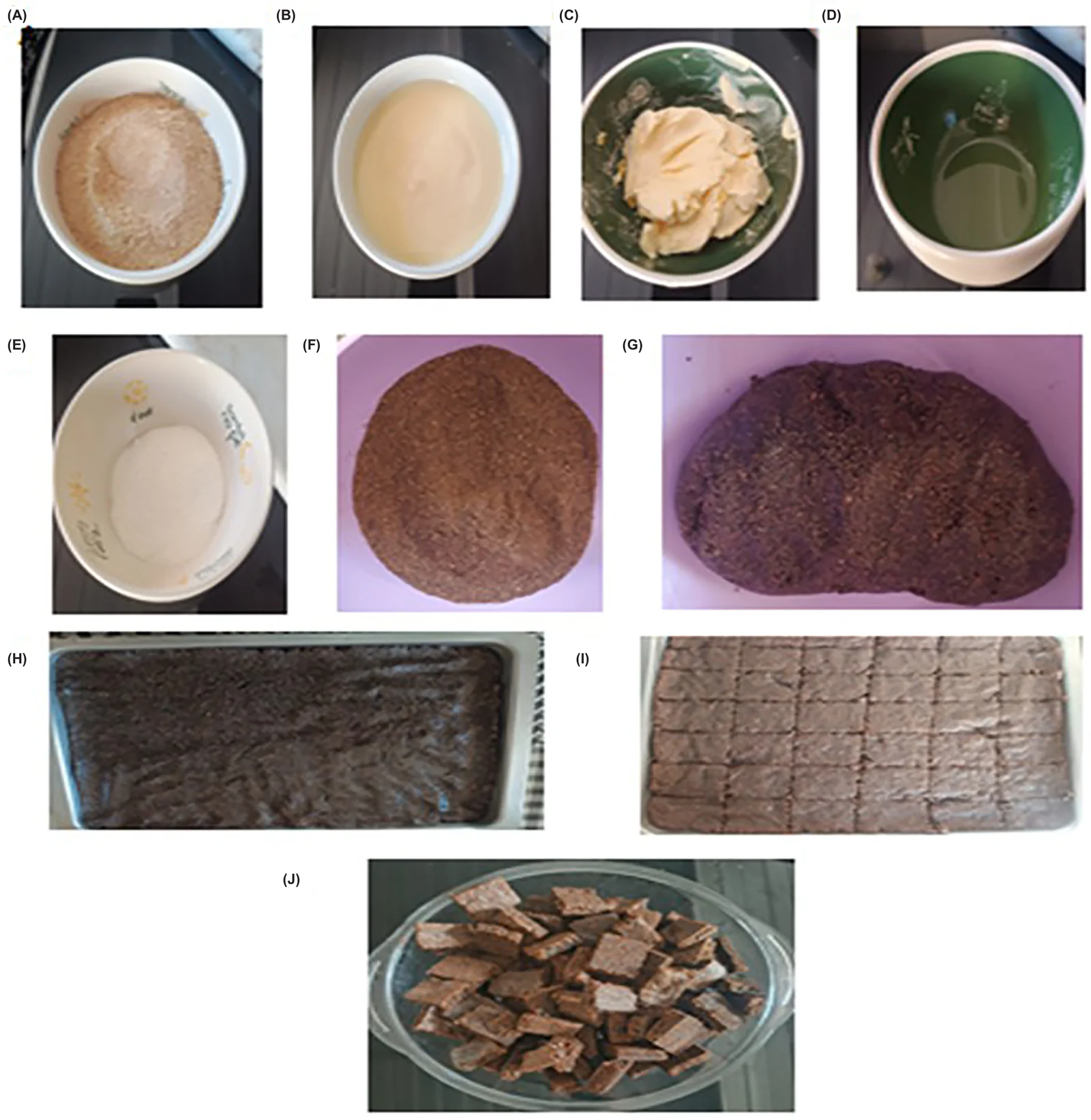
Individual Ingredients and Preparation of the Cafeteria Diet: Ingredient Identification, Homogenization, Baking, and Portioning for Animal Administration (**A**) Ground standard chow; (**B**) condensed milk; (**C**) margarine; (**D**) soybean oil; (**E**) refined sugar; (**F**) ultra-processed chocolate-filled biscuits; (**G**) homogenized mixture after incorporation of all ingredients. (**H**) The resulting dough was uniformly distributed on a greased tray (∼1 cm thickness) and thermally processed at 180°C for 30 min (10 + 20 min). (**I**,**J**) After baking, the material was portioned while warm into standardized cubes (∼3 × 3 × 1 cm) and cooled under controlled room conditions before administration to animals.

The resulting dough was evenly distributed onto a greased baking tray, spread into a uniform layer (approximately 1 cm thickness) to ensure even thermal exposure. The preparation was then subjected to thermal processing in a preheated oven at 180°C for a total of 30 min (initially for 10 min, followed by an additional 20 min).

This specific formulation accurately mimics the human “Western diet.” The thermal processing at 180°C, besides ensuring palatability and crunchiness, favors the formation of Maillard reaction products. These compounds significantly increase the diet’s attractiveness to the animals, promoting hedonic hyperphagia (overconsumption beyond caloric needs), which is essential for the rapid induction of the metabolic syndrome model.

After removal from the oven, the material was immediately portioned while still warm into square units with approximate dimensions of 3 × 3 × 1 cm (Figure 1). The portions were then maintained in a dry and ventilated environment under controlled room conditions until complete cooling. Food administration was standardized based on the total mass offered, measured using a precision balance at the time of feeding. This cafeteria diet was utilized throughout the experimental protocol to induce obesity, insulin resistance, and associated metabolic disturbances.

### Streptozotocin administration

STZ was prepared freshly under reduced light conditions and dissolved in citrate buffer (0.1 M, pH 4.5). The buffer was prepared using sodium citrate dihydrate and citric acid, with pH adjustment performed using HCl or NaOH. STZ was administered intraperitoneally at a dose of 35 mg/kg after 42 days of dietary intervention.

### Experimental timeline

- **Days 0–102:** Exposure to cafeteria or standard diet.
- **Day 42:** Saline or STZ administration i.p.
- **Day 102 (14 weeks):** Euthanasia and biological sample collection
  At the end of the experimental period (day 102; 14 weeks), animals were euthanized by anesthetic overdose using ketamine (240 mg/kg) and xylazine (30 mg/kg), administered to induce deep anesthesia followed by euthanasia, in accordance with institutional ethical guidelines and the approved Animal Ethics Committee (CEUA/UFRN) protocol. Adequate anesthetic depth was confirmed by the absence of reflex responses prior to sample collection.

Following euthanasia, blood samples were collected by cardiac puncture for hematological and biochemical analyses, as well as for quantification of circulating cytokines and insulin by enzyme-linked immunosorbent assay (ELISA)-based immunoassays. In addition, feces and organs were harvested for subsequent experimental analyses, including histopathological, molecular, inflammatory, and metabolic assessments.

### Anthropometric measurements and phenotypic classification

To evaluate the development of obesity and metabolic changes, anthropometric parameters were recorded at baseline (Day 1) and at the end of the experimental period (Day 102). Body weight and abdominal circumference were measured using a digital scale and a nonelastic tape measure, respectively.

The percentages of body weight gain and abdominal circumference gain were calculated using the formula:

$$\text{Gain }(\%)\;=\;\left(\frac{\left(\text{Final Value }-\text{ Initial Value}\right)}{\text{Initial Value}}\right)\times 100.$$

Animals were phenotypically classified based on weight gain thresholds: normal (<100%), overweight (100%–150%), and obese (>150%). For abdominal circumference, the criteria were: normal (<20%), overweight (20%–35%), and obese (>35%).

Additionally, the Lee index was calculated as an indicator of adiposity using the following equation:

$$\text{Lee Index }=\;\left(\frac{\text{root}(3,\text{Body Weight }(\text{g}))}{\text{Naso}-\text{anal Length }(\text{cm})}\right)\times 1000.$$

The classification for the Lee index followed established protocols: normal (<300), overweight (300–310), and obese (>310).

### Biochemical and hematological analyses

At the end of the experimental period (Day 102), blood samples were collected by cardiac puncture under anesthesia. Samples were divided into tubes with and without anticoagulant (EDTA) to obtain whole blood and serum, respectively.

A complete blood count was performed using an automated hematology analyzer. The parameters evaluated included: red blood cell count, hemoglobin, hematocrit, white blood cell count, and differential leukocyte counts (lymphocytes, monocytes, and segmented neutrophils). Platelet count and mean platelet volume were also recorded.

Serum was obtained by centrifugation at 3000 rpm for 15 min and stored at −20°C until analysis. The biochemical profile was determined using commercial kits (Bioclin, Mg, Brazil). The following markers were assessed:

- Glucose metabolism: Serum glucose and fructosamine.
- Lipid profile: Triglycerides, total cholesterol (CHOL), and high-density lipoprotein cholesterol.
- Renal Function: Urea, creatinine, and uric acid.
- Hepatic and muscle damage markers: Total protein, aspartate aminotransferase (AST), alanine aminotransferase (ALT), alkaline phosphatase, lactate dehydrogenase, and creatine kinase-MB.

The AST/ALT ratio was calculated as an indicator of liver health. All results were compared against established reference ranges for adult Wistar rats.

Glycated hemoglobin (HbA1c) levels were determined at the Clinical Analysis Laboratory of the University Hospital Onofre Lopes (HUOL), Federal University of Rio Grande do Norte (UFRN). HbA1c was determined in whole blood from Wistar rats using a commercial diagnostic kit (Cat. No. 417, Gold Analisa Diagnóstica Ltda., Belo Horizonte, MG, Brazil) based on ion-exchange chromatography. After hemolysis and removal of the labile fraction, stable HbA1c was separated by ion-exchange chromatography and quantified by spectrophotometry at 415 nm, according to the manufacturer’s instructions.Whole blood samples were collected at the end of the experimental period and analyzed to assess long-term glycemic control in the experimental groups. HbA1c values were interpreted comparatively relative to healthy control animals because universally validated diagnostic cutoffs for HbA1c in Wistar rats are not established. Therefore, classifications represent descriptive metabolic profiles rather than formal diagnostic categories of diabetes.

Because HbA1c measurement was performed in a subset of animals and aimed primarily at exploratory assessment of chronic glycemic status, these data were analyzed as complementary indicators of long-term metabolic disruption.

### Insulin and cytokine quantification by ELISA

Serum samples were analyzed for insulin, IL-1β, and IL-6 concentrations using ELISA using commercial ELISA kits for rats (R&D Systems), according to the manufacturer’s instructions. Insulin levels were quantified using a Rat Insulin ELISA Kit (Cloud-Clone Corp., CEA448Ra).

Blood samples were collected at the time of euthanasia and allowed to clot at room temperature. Samples were then centrifuged at approximately 1000 × ***g*** for 15–20 min, and serum was carefully collected, aliquoted, and stored at –80°C until analysis. Insulin concentrations in serum were measured using a competitive ELISA method. The assay was performed in 96-well microplates pre-coated with an insulin-specific antibody. Briefly, samples and standards were added to antibody-coated microplates, followed by incubation with enzyme-linked detection antibodies. After sequential washing steps, substrate solution was added, and the colorimetric reaction was developed and stopped as recommended. Absorbance was measured at 450 nm, and cytokine concentrations were calculated based on standard curves generated for each assay.

The Homeostatic Model Assessment (HOMA) was originally developed for humans using fasting glucose and insulin values expressed in μIU/ml. In rodents, HOMA indices are frequently used as relative metabolic markers; however, interpretation should be based on comparison with the healthy control group because no universal validated cutoff values exist for rats.

Insulin conversion: 1 μIU/ml ≈ 34.7 pg/ml

HOMA-IR = [Glucose (mg/dl) × Insulin (μIU/ml)]/405

HOMA-β = [360 × Insulin (μIU/ml)] / [Glucose (mg/dl) − 63]

### Gene expression analysis by RT-qPCR

Total RNA was extracted separately from visceral adipose tissue (mesenteric region) and skeletal muscle samples. Tissue samples were initially homogenized in TRIzol^®^ reagent according to the manufacturer’s instructions. Following the initial RNA extraction and phase separation, the RNA-containing fraction was further purified using the SV Total RNA Isolation System (Promega Corporation, Madison, WI, U.S.A.; Cat. No. Z3100), according to the manufacturer’s instructions. The RNA purification procedure included on-column DNase treatment to minimize genomic DNA contamination. RNA concentration and purity were assessed spectrophotometrically using a NanoDrop spectrophotometer. RNA concentrations varied among samples according to tissue and extraction yield, and the concentration obtained for each sample was recorded before cDNA synthesis. RNA purity was assessed using the A260/A280 and A260/A230 ratios.

For reverse transcription, up to 2 μg of total RNA from each sample was used for cDNA synthesis in a final reaction volume of 20 μl using the High-Capacity cDNA Reverse Transcription Kit (Applied Biosystems, Thermo Fisher Scientific, Waltham, MA, U.S.A.; Cat. No. 4368814), following the manufacturer’s instructions. The reverse transcription reaction was performed at 25°C for 10 min, 37°C for 120 min, and 85°C for 5 min, followed by storage at 4°C. The resulting cDNA was subsequently diluted before quantitative PCR.

Real-time PCR was performed using PowerUp SYBR Green Master Mix (Applied Biosystems, Thermo Fisher Scientific; Cat. No. A25742) in a StepOnePlus™ Real-Time PCR System (Applied Biosystems). Each 10-μl reaction contained 5 μl of SYBR Green Master Mix, 0.2 μl of forward primer, 0.2 μl of reverse primer, 2 μl of diluted cDNA, and nuclease-free water to the final reaction volume. A no-template control was included for each primer set.

The amplification protocol consisted of an initial denaturation at 95°C for 20 s, followed by 40 cycles of 95°C for 3 s and the primer-specific annealing temperature (56–60°C) for 30 s. A melting-curve analysis was performed at the end of each run to verify amplification specificity and to identify potential primer-dimer formation.

Gene expression was normalized to glyceraldehyde-3-phosphate dehydrogenase (GAPDH), which was used as the housekeeping/reference gene. Relative gene expression was calculated using the 2^−ΔΔCt^ method, with the corresponding control group used as the calibrator.

The specific target genes analyzed in adipose tissue and skeletal muscle, together with their respective primer sequences, are now explicitly indicated in Table 3.

**Table 3:** Primer sequences, target genes, and reference genes used for RT-qPCR analysis in adipose and skeletal muscle tissues of experimental male Wistar rats

Gene (specie)	Sequence
GAPDH	(Forward) 5′-AACTTGGCATCGTGGAAGG-3′
	(Reverse) 5′-GTGGATGCAGGGATGATGTTC-3′
Leptina Rat (Adipose tissue)	(Forward) 5′-GGAGGAATCCCTGCTCCAGC-3′
	(Reverse) 5′-CTTCTCCTGAGGATACCTGG-3′
*Slc2a4* (GLUT 4) Rat (skeletal muscle)	(Forward) 5′-GGGCTGTGAGTGAGTGCTTTC-3′
	(Reverse) 5′-CAGCGAGGCAAGGCTAGA-3′

### DNA extraction, quantification, and quality assessment

Genomic DNA was extracted from 250 mg of fecal samples using the QIAamp PowerFecal Pro DNA Kit (Qiagen), following the manufacturer’s protocol with bead-beating homogenization for 10 min to ensure efficient microbial cell lysis. DNA concentration and purity were assessed using both the Qubit 3.0 Fluorometer (Thermo Scientific) with the dsDNA BR Quantification Assay Kit and the NanoDrop One Microvolume UV-Vis Spectrophotometer (Thermo Scientific). DNA integrity was confirmed by 0.8% agarose gel electrophoresis mixed with SYBR Safe DNA Gel Stain 10 000× (Thermo Scientific). GeneRuler 100 bp Plus DNA Ladder (Thermo Scientific) was used as the molecular weight marker, the power supply was set to 110 V, and electrophoresis was performed for 1 h. The full uncropped agarose gel image used to assess DNA integrity is provided in the Supplementary Material (Supplementary Figure S1). Extracted DNA was stored at −20°C until further processing.

### Library preparation and nanopore sequencing

The library was assembled with Rapid Sequencing DNA-16S Barcoding Kit 24 V14 (SQK-16S114.24) (Oxford Nanopore Technologies). The genomic library was prepared using 10 ng of DNA adjusted to 15 μl with nuclease-free water, 25 μl of LongAmp Hot Start Taq 2× Master Mix (New England BioLabs), and 10 μl of barcoded primers under the PCR conditions in Table 4 (2 cycles). Reactions were stopped with 4 μl of EDTA, requantified via Qubit 3.0 Fluorometer (Thermo Scientific), and pooled in equimolar amounts.

**Table 4:** PCR amplification conditions and thermal cycling parameters used for genomic library preparation for gut microbiota sequencing in fecal samples from male Wistar rats

Cycle step	Temperature	Time	No. of cycles
Initial denaturation	95°C	1 min	1
Denaturation	95°C	20 s	25
Annealing	55°C	30 s	25
Extension	65°C	2 min	25
Final extension	65°C	5 min	2
Hold	4°C	∞	-

The pool was purified using AMPure XP Beads at a 0.6× ratio, washed twice with 80% ethanol, and eluted in 15 μl of elution buffer. Subsequently, 1 μl of pre-diluted Rapid Adapter was added to the eluate.

Sequencing was performed on a MinION MK1B device (Oxford Nanopore Technologies). The Flow Cell was primed with a mixture of Flow Cell Flush, BSA (50 mg/ml), and Flow Cell Tether according to the manufacturer’s instructions. Finally, a 75 μl loading library (comprising 37.5 μl Sequencing Buffer, 25.5 μl Library Beads, and 12 μl of the final DNA library) was loaded via the SpotON port, and sequencing was executed using MinKNOW software.

### Bioinformatic analysis

The run was terminated when each barcode reached a minimum of 30.000 reads. Taxonomic assignment was conducted using the Epi2me Labs 16S classification workflow, which maps reads against reference databases (NCBI 16S18S) for taxonomic identification down to genus level where possible. Raw sequencing reads were demultiplexed based on barcode sequences and quality-filtered using a minimum quality threshold (Q-score ≥7). Taxonomic profiles were expressed as relative abundance percentages using the mean data for each group.

Intra-sample microbial richness and evenness were estimated using Fisher alpha index (richness), the Shannon diversity index (richness and evenness), and the Simpson index (dominance weighting).

### Histopathological analysis

Histopathological analysis was performed on liver, kidney, pancreas, and adipose tissue samples obtained from the Healthy (Control) and Cafeteria groups. After euthanasia, organs were collected and fixed in 10% buffered formalin for 24–48 h. Subsequently, tissues were processed using standard histological techniques, embedded in paraffin, and sectioned at 4–5 μm thickness. Sections were stained with hematoxylin and eosin (H&E) for general morphological evaluation and examined using a 10× objective.

Histopathological alterations in the kidney, liver, pancreas, and adipose tissue were assessed using semiquantitative scoring systems based on the severity or extent of the observed lesions. The evaluated parameters and their respective scoring criteria are summarized in Table 5. Scores ranged from 0 to 4, with higher scores indicating greater severity or extent of the histopathological alteration. The renal criteria were based on previously established histopathological evaluation approaches [16–18]. whereas hepatic, pancreatic, and adipose tissue parameters were adapted from previously described scoring systems [19–21].

**Table 5:** Semiquantitative scoring criteria used for histopathological evaluation of the organs

Organ	Histopathological parameter	Score 0	Score 1	Score 2	Score 3	Score 4
**Kidney**	Glomerulosclerosis	Normal	<25% of glomeruli affected	25%–50%	50%–75%	>75%
	Interstitial fibrosis	Absent	Minimal	Mild	Moderate	Severe
	Arteriolar hyalinosis	Absent	Mild	Moderate	Marked	Severe
	Inflammatory infiltration	Absent	<25% of field	25%–50%	50%–75%	>75%
	Tubular alterations	Absent	Minimal	Mild	Moderate	Severe
**Liver**	Hepatic steatosis	Absent	<25%	25%–50%	50%–75%	>75%
	Lobular inflammation	Absent	Minimal	Mild	Moderate	Severe
	Hepatocellular ballooning	Absent	Minimal	Mild	Moderate	Severe
	Sinusoidal congestion	Absent	Mild	Moderate	Marked	Severe
**Pancreas**	Islet integrity	Normal structure	Minimal alterations	Mild cellular disorganization	Moderate disorganization	Severe disruption/loss
	Inflammatory infiltration	Absent	Minimal	Mild	Moderate	Severe
	Cellular degeneration	Absent	Minimal	Mild	Moderate	Severe
**Adipose tissue**	Adipocyte hypertrophy	Normal size	Minimal hypertrophy	Mild	Moderate	Severe
	Inflammatory infiltration	Absent	Minimal	Mild	Moderate	Severe
	Adipose tissue fibrosis	Absent	Minimal	Mild	Moderate	Severe

Note: Scores ranged from 0 to 4, with higher scores indicating greater severity or extent of the histopathological alteration. The total renal and hepatic scores were calculated by summing the scores assigned to the respective evaluated parameters.

### Statistical analysis

Data were analyzed according to their distribution. Continuous variables were expressed as median and interquartile range (IQR; 25th–75th percentiles). Nonparametric data were analyzed using the Kruskal–Wallis test followed by Dunn’s post hoc test for comparisons among more than two groups, or the Mann–Whitney U test for comparisons between two groups. Statistical significance was set at *P* < 0.05.

## Results

### Anthropometrics, hematological, and biochemistry

Body weight gain was calculated considering the body weight measured on day 1 as 100%, and the percentage variation was determined after 102 days of the experimental protocol. The same calculation approach was applied to abdominal circumference measurements.

Animals fed the standard diet showed moderate physiological growth during the experimental period. The Standard Diet + Saline group exhibited an average body weight increase of 81.7%, whereas the Standard Diet + STZ group showed a similar increase of 78.3%. Abdominal circumference increased by 13.1% and 17.3%, respectively, remaining within the predefined range for normal growth according to the anthropometric criteria used in the present study (Table 6).

**Table 6:** Anthropometric parameters, obesity phenotype classification, and adiposity indices in male Wistar rats

Group	Body weight gain (%)^a^	Abdominal circumference gain (%)^b^	Metabolic classification^a,b^	Lee index (102 days)	Classification on last day by Lee index^c^
Health	+81.7%	+13.1%	Normal	295.4	Normal
STZ	+78.3%	+17.3%	Normal	296.7	Normal
Cafeteria+ STZ	+131.4%	+31.8%	Overweight	296.9	Normal
Cafeteria	+172.3%	+38.7%	Obese	304.6	Overweight

a Phenotype/weight gain: <100% (Normal); 100%–150% (Overweight); >150% (Obese).

b Phenotype/abdominal circumference: <20%(Normal); 20%–35%(Overweight); >35%(Obese).

c <300 (Normal); 300–310 (Overweight); >310 (Obese).

In contrast, animals receiving the cafeteria diet exhibited substantially greater increases in body weight and abdominal circumference. The Cafeteria Diet + STZ group showed a 131.4% increase in body weight and a 31.8% increase in abdominal circumference, consistent with an overweight phenotype according to the predefined anthropometric criteria (Table 6). The Cafeteria Diet group exhibited the most pronounced anthropometric changes, with a 172.3% increase in body weight and a 38.7% increase in abdominal circumference, corresponding to an obese phenotype according to the same criteria (Table 6).

Overall, these findings support the obesogenic effect of the cafeteria diet based on the marked increases in body weight and abdominal circumference. Interestingly, the Cafeteria Diet + STZ group showed lower increases in both parameters than the Cafeteria Diet group, suggesting that STZ administration partially attenuated the weight gain and abdominal expansion induced by the cafeteria diet. This finding may be related to the metabolic effects of STZ, including impaired pancreatic β-cell function and reduced insulin availability, which may promote a more catabolic state and limit body-weight gain despite the continued exposure to a high-calorie diet. Importantly, the anthropometric findings presented in Table 6 should be interpreted independently from the biochemical and metabolic alterations assessed in other analyses of the study.

The Lee index increased during the experimental period in all groups, consistent with normal growth and changes in body size. At day 102, the Cafeteria Diet group presented the highest Lee index among the experimental groups, reaching a value of 304.6, which corresponds to the overweight range according to the predefined cutoff used in the present study. In contrast, the Cafeteria Diet + STZ group presented a Lee index of 296.9, remaining within the normal range (<300), despite exhibiting an overweight phenotype based on body weight gain and abdominal circumference. The Standard Diet + Saline and Standard Diet + STZ groups also remained within the normal range, with Lee index values of 295.4 and 296.7, respectively (Table 6). These findings further support the interpretation that the Lee index should be considered as a complementary anthropometric indicator rather than as a stand-alone criterion for defining obesity.

In the STZ group, triglyceride levels were lower (median ≈ 96 mg/dl) but highly variable, indicating that STZ-induced diabetes alone did not consistently increase triglyceride concentrations. Animals fed a cafeteria diet displayed higher triglyceride levels (median ≈ 131 mg/dl), indicating an alteration in triglyceride concentrations associated with the hypercaloric diet. Notably, the Cafeteria + STZ group showed the highest triglyceride levels (median ≈ 145 mg/dl), together with a broader dispersion of values, suggesting an exacerbated and heterogeneous metabolic response. These findings indicate that the combination of a hypercaloric diet and STZ was associated with greater changes in triglyceride concentrations than either intervention alone, while also highlighting the biological variability inherent to experimental animal models (Table 7 and Supplementary Table S1).

**Table 7:** Serum biochemical markers of glucose metabolism, lipid profile, renal function, and hepatic injury in male Wistar rats

Groups	Glucose Maior que 300 mg/dl	Fructosamine 40–140 (μmol/l)	TG 23–87 (mg/dl)	CHOL 34–70 (mg/dl)	HDL-C 14–31 (mg/dl)	UREA 34–72 (mg/dl)	CREATININE 0.30–0.60 (mg/dl)	UA 0.9–1.5 (mg/dl)	TP 5.4–7.2 (g/dl)	AST 37–196 (U/l)	ALT 29–78 (U/l)	AST/ALT ratio 0.5–6.8 (U/l)	ALP 88–164 (U/l)	LDH 380–1587 (U/l)	CK-MB 5.9–86.6 (U/l)
**HEALTHY**	251 (231–276)	143 (139–145)	64 (55–70.5)	63 (54–65)	60 (53–65)	38 (35–42)	1 (1–1)	1 (1–5)	6 (6–6)	95 (86–124)	60 (57–88)	1 (1–2)	170 (159–176)	387 (326–476	230 (134–365)
**STZ**	284 (270–305)	111 (100–157)	96 (76–148	58 (50–59)	70 (64–76)	58 (42–62	1 (1–1)	3 (2–4)	6 (5–6)	113 (108–115)	52 (37–66)	2 (2–2)	133 (124–136)	1009 (445–1101)	325 (324–406)
**CAFETERIA + STZ**	388 (326–472)	122 (107–194)	145 (112–193	58 (53–61)	55 (48–60)	35 (32–39)	1 (1–1)	2 (2–3)	6 (6–6)	99 (82–122	32 (27–56)	3 (2–4)	181 (162–229)	757 (441–931)	402 (309–533)
**CAFETERIA**	403 (370–529	120 (106–156)	131 (111–154)	60 (55–66)	54 (51–58)	32 (30–73)	1 (1–1)	1 (1–2)	6 (6–6)	78 (77–89	52 (47–68	2 (1–2)	250 (203–324)	676 (461–784)	342 (291–495)

Values of glucose, fructosamine, triglycerides (TG), total cholesterol (CHOL), HDL-cholesterol (HDL-C), urea, creatinine, uric acid (UA), total protein (TP), aspartate aminotransferase (AST), alanine aminotransferase (ALT), AST/ALT ratio, alkaline phosphatase (ALP), lactate dehydrogenase (LDH), and CK-MB in the Healthy (control), STZ, Cafeteria + STZ, and Cafeteria groups. Data are presented as median (interquartile range). Units are indicated for each parameter. The STZ group represents animals with streptozotocin-induced diabetes, the Cafeteria group represents animals fed a cafeteria diet, and the Cafeteria + STZ group represents animals exposed to both the cafeteria diet and STZ-induced diabetes.

Renal biochemical parameters, including urea, uric acid, and creatinine, remained within the reference values established for rats, with no evidence of clinically relevant renal dysfunction based on these parameters alone (Table 7 and Supplementary Table S1).

Total protein concentrations remained within the normal range in all experimental groups, indicating the absence of systemic protein loss, malnutrition, or significant impairment of hepatic synthetic function (Table 7 and Supplementary Table S1).

AST and ALT levels remained within reference ranges in most groups, suggesting the absence of extensive hepatocellular necrosis. The AST/ALT ratio remained within physiological limits, supporting relative stability of hepatocellular integrity (Table 7 and Supplementary Table S1).

HbA1c levels were measured in animals from all experimental groups at the end of the experimental period to provide a complementary assessment of long-term glycemic status. Most male Wistar rats from the Healthy, STZ, Cafeteria, and Cafeteria + STZ groups exhibited HbA1c values within the physiological range observed in healthy controls (approximately 3.9%–4.6%), indicating no marked chronic glycemic alteration under the experimental conditions (Supplementary Table S2).

Notably, a small subset of animals from the Cafeteria and Cafeteria + STZ groups exhibited markedly elevated HbA1c values relative to healthy controls, including values of 9.5% and 8.7% in the Cafeteria group and 8.1% in the Cafeteria + STZ group (Supplementary Table S2). These findings suggest interindividual variability in metabolic susceptibility, with some animals developing a profile compatible with more pronounced chronic glycemic dysregulation following prolonged cafeteria diet exposure. Importantly, because validated diagnostic HbA1c cutoffs for Wistar rats are not universally established, these findings should be interpreted comparatively relative to healthy control animals rather than as formal diagnostic evidence of diabetes. The complete individual dataset is presented in Supplementary Table S2.

A lymphocyte-predominant leukocyte profile was observed across all groups, which is expected in rodents. However, the Cafeteria + STZ group showed greater leukocyte variability, suggesting a chronic metabolic and inflammatory impact rather than systemic infection (Table 8 and Supplementary Table S3).

**Table 8:** Hematological profile of male Wistar rats

PARAMETERS	RBC 5.2–9.6 (10^6^/μl)	HGB 10.9–17.1 (g/ml)	HCT 30–55 (%)	WBC 2.6–13.8 (10^3^/μl)	LYM 70–80 (10^3^/μl)	MONO 0–4 (%)	NEU-SEG 35–71 (%)	PLT 745–1436 (10^3^/μl)	MPV 5.2–6.6 (fl)
**HEALTHY**	8.12 (7.80–8.64)	15.75 (14.40–16.65)	44.05 (41.53–46.85)	3.25 (2.45–4.50)	76.00 (72.50–79.00)	1.00 (1.00–1.00)	18.50 (12.75–20.50)	893.00 (728.00–927.50)	4.80 (4.65–4.85)
**STZ**	8.20 (7.99–8.74)	14.90 (14.70–16.18)	43,20 (42.90–46.88	3.10 (3.00–3.25	74.00 (74.00–76.25)	1.00 (0.00–1.00)	18.00 (15.00–21.00)	949.00 (932.00–1079.50)	4.50 (4.50–4.50)
**CAFETERIA + STZ**	8.52 (8.12–8.88)	15.85 (15.05–16.50)	44.95 (44.38–48.70)	4.70 (3.88–5.10)	81.50 (72.75–83.50)	1.00 (1.00–2.00)	15.50 (11.00–18.50)	877.00 (805.00–987.50	4.85 (4.60–5.35)
**CAFETERIA**	8.44 (8.37–8.68	15.60 (15.10–16.00)	45.40 (45.10–46.00)	4.70 (4.25–4.90)	76.00 (72.00–85.50)	0.00 (0.00–0.00)	21.00 (12.00–22.25)	921.00 (861.00–961.00)	4.50 (4.45–4.75)

Data are presented as median (interquartile range). Reference values for each parameter are shown in the column headers. RBC: red blood cells; HGB: hemoglobin; HCT: hematocrit; WBC: total leukocytes; LYM: lymphocytes; MONO: monocytes; NEU-SEG: segmented neutrophils; PLT: platelets; MPV: mean platelet volume. STZ: streptozotocin-induced diabetes; CAFETERIA: animals fed a cafeteria diet.

Animals in the healthy control group presented a mean glucose concentration of 254.83 ± 44.26 mg/dl, whereas the STZ-treated group exhibited a similar mean glycemic concentration of 257.20 ± 105.83 mg/dl (Supplementary Table S4). Although the STZ group showed greater interindividual variability, as indicated by the relatively high standard deviation, the mean glucose concentration was not markedly different from that observed in the healthy control group. Therefore, these data alone do not support a conclusion of pronounced hyperglycemia in the STZ group.

In contrast, the STZ + Cafeteria and cafeteria groups exhibited higher mean glucose concentrations, reaching 391.21 ± 105.25 and 452.29 ± 126.66 mg/dl, respectively (Supplementary Table S4). These findings indicate greater disturbances in glucose homeostasis in the groups exposed to the cafeteria diet, particularly in the Cafeteria group. However, because glucose measurements were obtained during euthanasia under ketamine/xylazine anesthesia, the absolute glucose concentrations should be interpreted cautiously. Xylazine may induce transient hyperglycemia through α2-adrenergic receptor-mediated suppression of insulin secretion. Thus, the measured glucose concentrations may have been partially influenced by the anesthetic protocol. Since all experimental groups underwent the same anesthesia and euthanasia procedures, this potential effect was considered systematic and does not preclude comparative interpretation among groups.

Serum insulin concentrations were 5748.97 ± 1339.76 pg/ml in the healthy control group, 7478.40 ± 2164.66 pg/ml in the STZ group, 7755.21 ± 1882.86 pg/ml in the STZ + cafeteria group, and 7061.49 ± 700.47 pg/ml in the Cafeteria group (Supplementary Table S4). Although the STZ and STZ + Cafeteria groups showed numerically higher insulin concentrations than the healthy control group, the substantial variability within groups should be considered when interpreting these differences. Accordingly, these data were considered together with the HOMA indices rather than interpreted independently.

To facilitate comparative interpretation, HOMA-IR values were considered relative to the healthy control group, which presented a mean value of 105.88 ± 38.71. The STZ group exhibited a mean HOMA-IR of 122.79 ± 59.72, whereas higher values were observed in the STZ + Cafeteria and Cafeteria groups, with means of 198.67 ± 54.92 and 229.19 ± 73.02, respectively (Supplementary Table S4). These results indicate a progressive numerical increase in HOMA-IR in the groups exposed to the cafeteria diet, particularly in the CAFETERIA group.

Importantly, HOMA-IR values were statistically compared among the experimental groups. One-way ANOVA demonstrated a significant overall difference among groups (F = 6.054, *P* = 0.0015), followed by Tukey’s post-hoc test. HOMA-IR was significantly higher in the Cafeteria group than in the Healthy group (*P* = 0.0053) and the STZ group (*P* = 0.0448). In addition, the STZ + Cafeteria group exhibited significantly higher HOMA-IR than the Healthy group (*P* = 0.0268). These findings support the presence of greater insulin resistance in the groups exposed to the cafeteria diet, particularly when compared with the healthy control and STZ groups.

Given the known effects of ketamine/xylazine anesthesia on glucose metabolism and the absence of universally validated diagnostic cutoffs for HOMA-IR in Wistar rats, these indices were interpreted as comparative markers of metabolic alteration rather than as absolute diagnostic thresholds for insulin resistance.

HOMA-β values also differed numerically among groups. The healthy control group exhibited a mean HOMA-β of 319.25 ± 30.65, whereas the STZ group presented a markedly higher and highly variable value of 1941.06 ± 1612.04. The STZ + Cafeteria and Cafeteria groups showed mean values of 319.78 ± 69.73 and 201.71 ± 18.52, respectively. These findings suggest substantial heterogeneity in the estimated β-cell functional response, particularly following STZ administration. However, because HOMA-β is an indirect index and was not statistically analyzed as a primary outcome, these values were interpreted descriptively and used as complementary information rather than as direct evidence of preserved or impaired pancreatic β-cell function.

Overall, the updated results indicate that exposure to the cafeteria diet was associated with higher glucose concentrations and significantly increased HOMA-IR values, particularly in the Cafeteria and STZ + Cafeteria groups. These findings are consistent with altered glucose homeostasis and increased insulin resistance associated with the hypercaloric dietary intervention. Nevertheless, the data do not by themselves establish a diagnosis of type 2 diabetes or allow definitive classification of disease severity. The HOMA-β results further demonstrate variability in the estimated β-cell response among groups and should therefore be interpreted as complementary evidence of metabolic adaptation rather than as a direct measure of pancreatic β-cell function.

### Cytokines and qRT-PCR

The experimental models (STZ, Cafeteria + STZ, and Cafeteria) induced alterations in circulating inflammatory markers and tissue-specific gene expression compared with the Healthy control group. Regarding low-grade systemic inflammation, both IL-1β and IL-6 serum levels were significantly elevated in the experimental groups compared with the Healthy group. Specifically, a marked increase in IL-1β was observed in the Cafeteria + STZ and Cafeteria groups (*P* < 0.05), suggesting an enhanced inflammatory response associated with the hypercaloric dietary intervention (Figure 2A). Similarly, IL-6 levels were significantly increased in the Cafeteria + STZ and Cafeteria groups (*P* < 0.05), supporting the presence of low-grade systemic inflammation associated with the metabolic alterations induced by the experimental interventions (Figure 2B).

**Figure 2:**
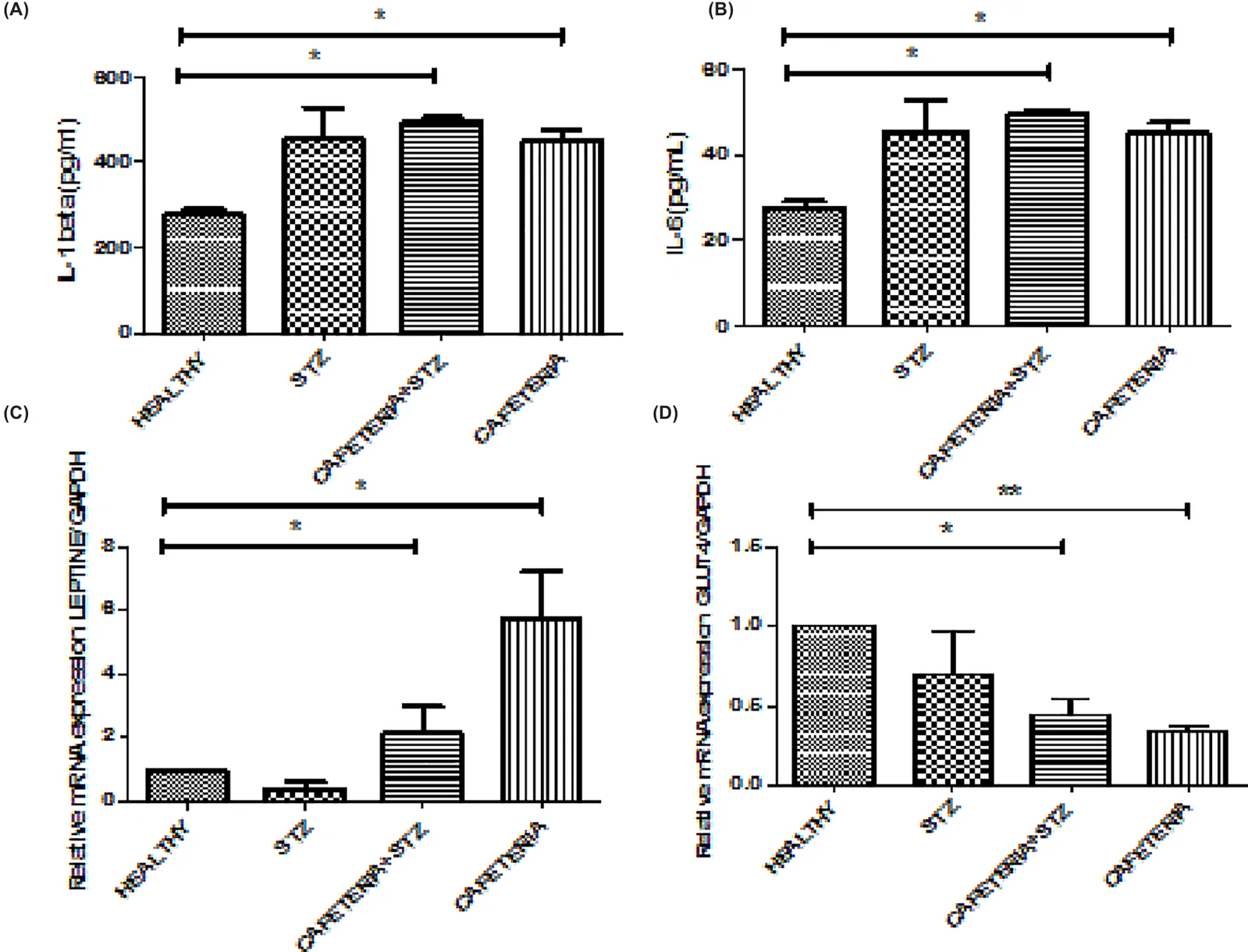
Inflammatory Cytokine Levels and Metabolic Gene Expression in Male Wistar Rats After 102 Days of Experimental Intervention Serum concentrations of IL-1β and IL-6 and relative gene expression of adipose tissue *Leptin* and skeletal muscle *Slc2a4* (GLUT4) in male Wistar rats allocated to the Healthy Control, STZ, Cafeteria, and Cafeteria + STZ groups after 102 days of experimental intervention. Data are expressed as mean ± standard deviation. Statistical analysis was performed using one-way ANOVA followed by Tukey’s post hoc test. Significant differences are indicated in the figure (*P < 0.05; **P < 0.01). (**A**) Serum IL-1β concentration; (**B**) serum IL-6 concentration; (**C**) relative *Slc2a4* (GLUT4) mRNA expression in skeletal muscle; (**D**) relative *Leptin* mRNA expression in adipose tissue.

Relative mRNA expression of *Leptin* in adipose tissue was significantly up-regulated in the Cafeteria and Cafeteria + STZ groups (*P* < 0.05) compared with the Healthy control group. Notably, the Cafeteria-only group exhibited the highest fold change, which may reflect the increased adipose tissue mass associated with the hypercaloric diet (Figure 2D). Conversely, significant down-regulation of *Slc2a4* (GLUT4) mRNA expression in skeletal muscle was observed in the Cafeteria + STZ (*P* < 0.05) and Cafeteria groups (*P* < 0.01) compared with the Healthy group. The reduced *Slc2a4* (GLUT4) expression in these groups suggests impaired glucose transport capacity in skeletal muscle and is consistent with an insulin-resistant metabolic phenotype (Figure 2C).

### Gut microbiota analysis

The analysis of the intestinal microbiota in the Cafeteria group revealed a distinct microbial profile associated with cafeteria diet exposure, characterized by marked taxonomic dominance despite relatively preserved alpha diversity. The Shannon index was higher in the Cafeteria group than in the Health group (2.12 ± 0.72 versus 1.73 ± 0.19), whereas the Simpson index was comparable between groups (0.638 ± 0.197 versus 0.633 ± 0.064). The Fisher index was slightly lower in the Cafeteria group than in the Health group (50.84 ± 3.87 versus 52.12 ± 2.08). Overall, these findings do not indicate a generalized loss of within-sample diversity in the Cafeteria group. Rather, the observed microbiota profile appears to be characterized predominantly by changes in community composition and taxonomic dominance (Table 9A and Figure 3A).

**Figure 3:**
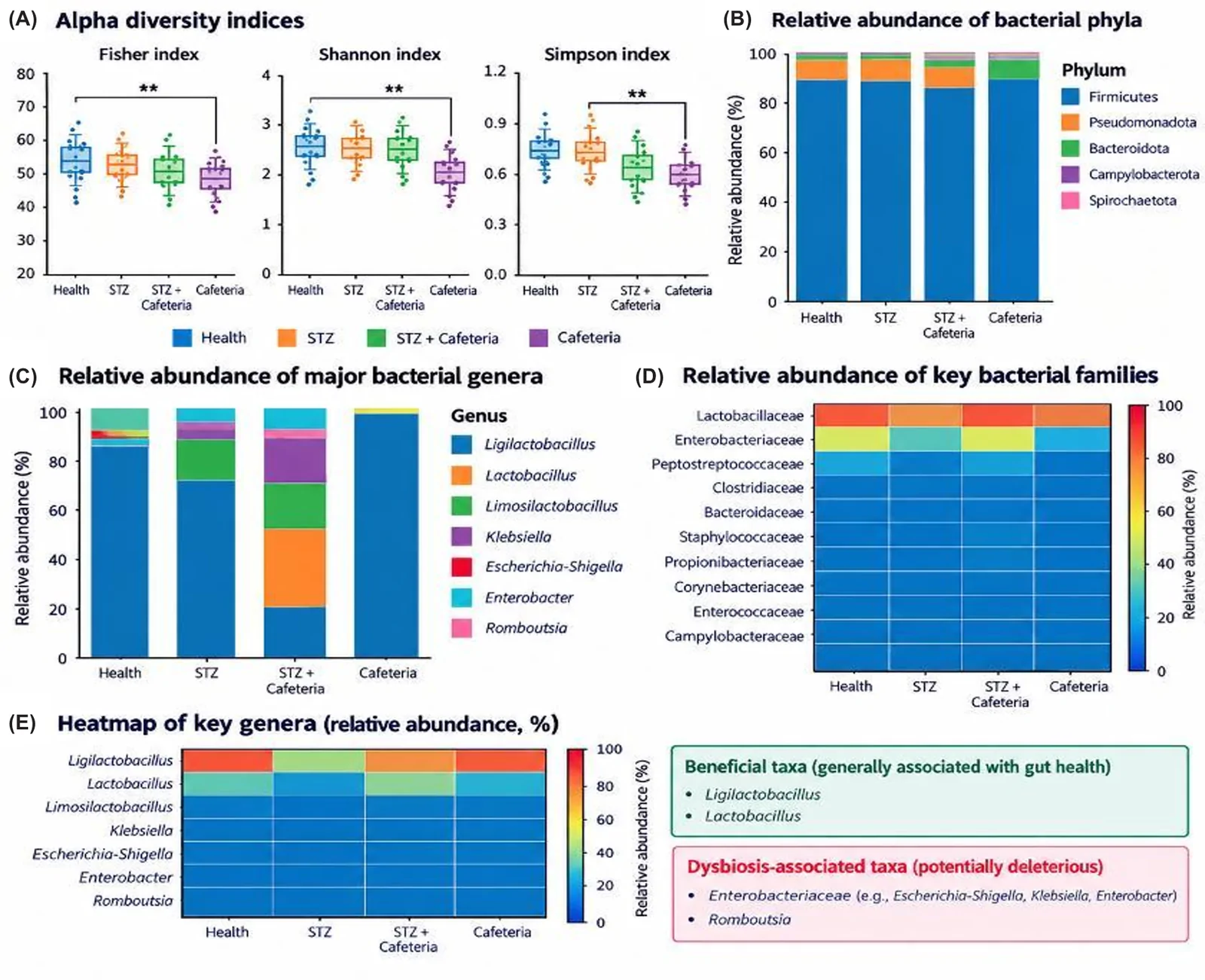
Gut microbiota diversity and composition across the experimental groups (**A**) Alpha-diversity indices, including Fisher, Shannon, and Simpson indices, comparing Health, STZ, STZ + Cafeteria, and Cafeteria groups. (**B**) Relative abundance of the predominant bacterial phyla. (**C**) Relative abundance of the major bacterial genera, including *Ligilactobacillus, Lactobacillus, Limosilactobacillus, Klebsiella, Escherichia-Shigella, Enterobacter*, and *Romboutsia*. (**D**) Relative abundance of selected bacterial families. (**E**) Heatmap showing the relative abundance of key bacterial genera across experimental groups. The Cafeteria group exhibited a distinct reduction in alpha-diversity, particularly in Fisher, Shannon, and Simpson indices, together with marked shifts in bacterial composition, characterized by changes in the relative abundance of major bacterial taxa. Values are expressed as relative abundance (%). The color scale in the heatmaps represents relative abundance, from low (blue) to high (red). Health, healthy control; STZ, streptozotocin; STZ + Cafeteria, streptozotocin combined with cafeteria diet; Cafeteria, cafeteria diet. Asterisks indicate statistically significant differences according to the statistical analysis described in the Methods (*P* < 0.05; *P* < 0.01).

**Table 9:** Relative abundance of gut bacterial taxa identified by Nanopore sequencing in fecal samples from male Wistar rats subjected to cafeteria diet-induced metabolic dysfunction

A: Alpha diversity indices across experimental groups				
Diversity index	Health	STZ	Cafeteria + STZ	Cafeteria
Fisher	52.12 ± 2.08	49.18 ± 6.89	51.73 ± 4.00	50.84 ± 3.87
Shannon	1.73 ± 0.19	2.89 ± 0.37	2.72 ± 0.52	2.12 ± 0.72
Simpson	0.633 ± 0.064	0.893 ± 0.025	0.848 ± 0.056	0.638 ± 0.197

B: Relative abundance of bacterial phyla (%)				
Phylum	Health	STZ	Cafeteria + STZ	Cafeteria
Firmicutes	96.37	93.56	86.61	99.83
Pseudomonadota	3.62	6.36	12.95	0.14
Bacteroidota	0.00	0.08	0.40	0.03
Campylobacterota	0.01	0.01	0.03	0.00
Spirochaetota	0.00	0.00	0.01	0.00

C: Relative abundance of major bacterial genera (%)				
Genus	Health	STZ	Cafeteria + STZ	Cafeteria
*Ligilactobacillus*	90.41 ± 9.51	95.16 ± 5.79	71.79 ± 27.01	99.36 ± 0.61
*Lactobacillus*	0.06 ± 0.11	0.16 ± 0.28	15.46 ± 15.11	0.30 ± 0.67
*Limosilactobacillus*	9.14 ± 9.31	0.00 ± 0.00	0.00 ± 0.00	0.00 ± 0.00
*Klebsiella*	0.00 ± 0.00	0.00 ± 0.00	5.21 ± 10.15	0.00 ± 0.00
*Escherichia-Shigella*	0.00 ± 0.00	0.00 ± 0.00	4.66 ± 11.42	0.00 ± 0.00
*Enterobacter*	0.00 ± 0.00	0.00 ± 0.00	2.33 ± 5.72	0.00 ± 0.00
*Romboutsia*	0.00 ± 0.00	3.63 ± 6.28	0.00 ± 0.00	0.00 ± 0.00

D: Relative abundance of key bacterial families (%)				
Family	Health	STZ	Cafeteria + STZ	Cafeteria
Lactobacillaceae	99.62 ± 0.33	91.38 ± 11.05	85.72 ± 15.45	99.52 ± 0.44
Enterobacteriaceae	0.00 ± 0.00	4.37 ± 5.79	13.73 ± 14.96	0.14 ± 0.12
Peptostreptococcaceae	0.00 ± 0.00	3.23 ± 5.59	0.00 ± 0.00	0.00 ± 0.00
Clostridiaceae	0.00 ± 0.00	0.73 ± 1.26	0.00 ± 0.00	0.00 ± 0.00
Bacteroidaceae	0.00 ± 0.00	0.05 ± 0.09	0.51 ± 0.65	0.02 ± 0.03
Staphylococcaceae	0.02 ± 0.02	0.12 ± 0.19	0.00 ± 0.00	0.31 ± 0.35
Propionibacteriaceae	0.19 ± 0.33	0.00 ± 0.00	0.00 ± 0.00	0.00 ± 0.00
Corynebacteriaceae	0.17 ± 0.29	0.00 ± 0.00	0.00 ± 0.00	0.00 ± 0.00
Enterococcaceae	0.00 ± 0.00	0.13 ± 0.22	0.00 ± 0.00	0.00 ± 0.00
Campylobacteraceae	0.00 ± 0.00	0.00 ± 0.00	0.05 ± 0.12	0.00 ± 0.00

At the phylum level, the Cafeteria group exhibited an extreme predominance of Firmicutes (99.83%), with only minimal representation of Pseudomonadota (0.14%) and Bacteroidota (0.03%) (Table 9B and Figure 3B). This pronounced distribution indicates substantial restructuring of the gut microbial community following cafeteria diet exposure. However, because relative abundance data do not directly demonstrate microbial function, these findings should be interpreted as evidence of altered microbial composition rather than definitive evidence of impaired metabolic flexibility, gut resilience, or other functional consequences.

At the genus level, the Cafeteria group showed an overwhelming predominance of *Ligilactobacillus* (99.36 ± 0.61%), with only minor contributions from *Lactobacillus* (0.30 ± 0.67%) and other genera (Table 9C and Figure 3C,E). Although lactobacilli are frequently associated with potentially beneficial functions, the pronounced dominance of a single genus may reflect reduced community evenness and a more specialized microbial structure. Importantly, this compositional pattern should not be interpreted as evidence of either a protective or detrimental effect without functional or mechanistic analyses.

In contrast with the STZ + Cafeteria group, the Cafeteria group showed no substantial expansion of genera such as *Klebsiella*, *Escherichia-Shigella*, or *Enterobacter*, which were more represented in the combined STZ + Cafeteria condition (Table 9C and Figure 3C,E). Similarly, Enterobacteriaceae represented only 0.14% of the Cafeteria microbiota, compared with 13.73% in the STZ + Cafeteria group (Table 9D and Figure 3D). These findings indicate that cafeteria diet exposure alone was associated with a distinct microbial restructuring without the pronounced enrichment of these taxa observed when cafeteria diet was combined with STZ treatment.

Family-level analysis further supported the marked dominance of Lactobacillaceae in the Cafeteria group (99.52%), whereas Enterobacteriaceae accounted for only 0.14% (Table 9D and Figure 3D). The heatmap and abundance profiles provide a complementary visualization of these compositional differences across experimental groups, highlighting the predominance of *Ligilactobacillus* in the Cafeteria group and the greater representation of several taxa within the Enterobacteriaceae family in the STZ + Cafeteria group (Figure 3C–E).

Collectively, these findings indicate that the Cafeteria group exhibited a pronounced alteration in gut microbial composition, characterized primarily by strong taxonomic dominance and marked shifts in community structure rather than a generalized reduction in alpha diversity. The absence of substantial expansion of Enterobacteriaceae and related genera distinguishes the Cafeteria group from the STZ + Cafeteria condition. These findings may represent an early or compositionally distinct stage of microbiota alteration associated with cafeteria diet exposure. However, functional consequences, including changes in short-chain fatty acid production, gut barrier integrity, or systemic endotoxemia, cannot be established from relative abundance data alone.

### Histopathological

Histopathological evaluation of the liver, kidneys, and pancreas revealed no statistically significant differences among the experimental groups. In the liver, the Cafeteria group showed focal portal and lobular mononuclear inflammatory infiltrates and mild disorganization of hepatocyte cords, whereas no statistically significant difference in the overall histopathological score was observed compared with the Healthy group (*P* > 0.05). Steatosis was not observed in the Cafeteria group, Figure 4.

**Figure 4:**
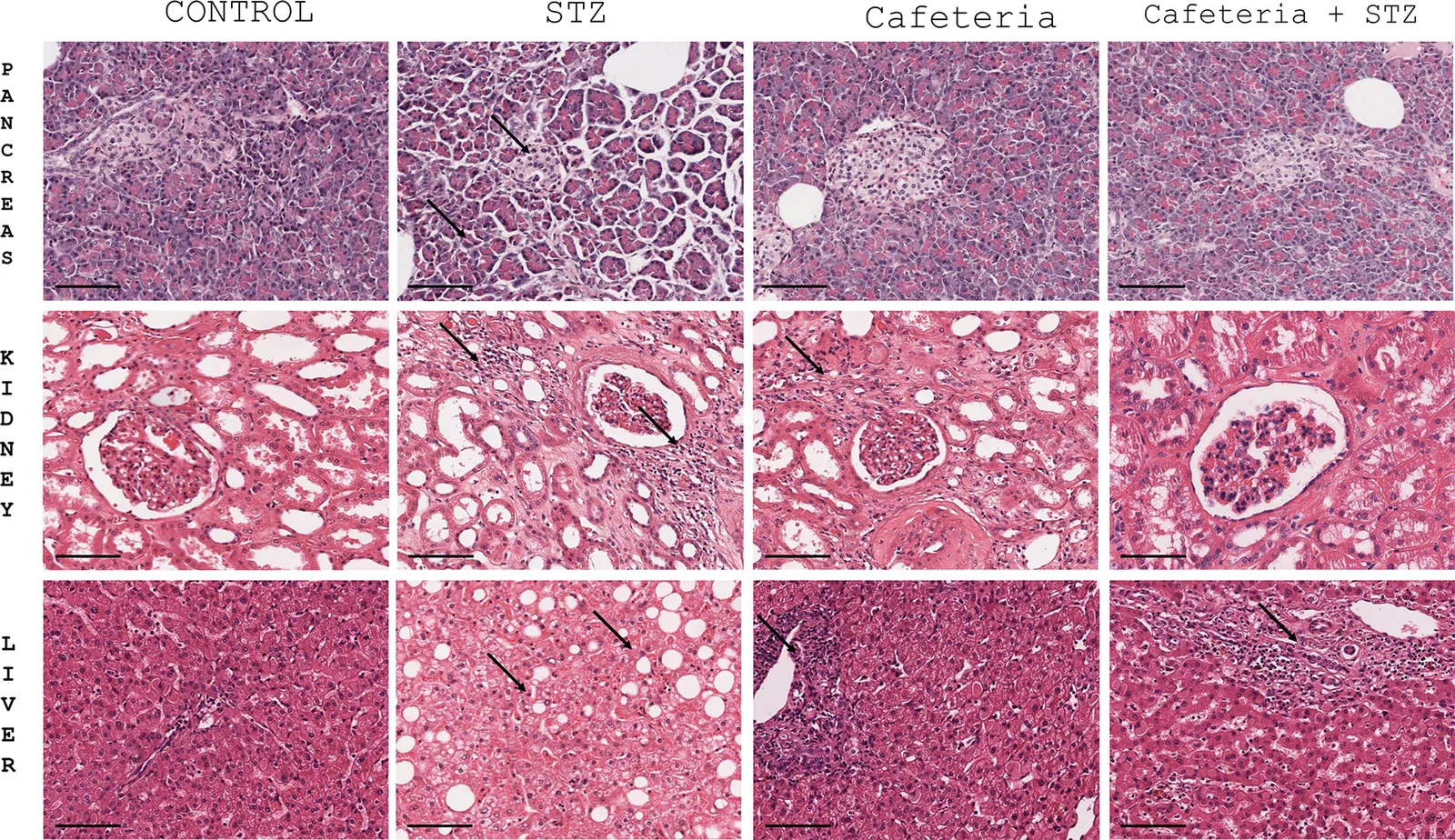
Histopathological changes in the pancreas, kidney, and liver of rats with STZ-induced diabetes and/or fed a cafeteria diet Sections stained with H&E. *Pancreas (upper row):* the CONTROL group shows islets of Langerhans with preserved size and cellularity, surrounded by intact acinar parenchyma; the STZ group shows atrophic islets with irregular outlines and reduced cellularity (arrows), together with retraction of the endocrine tissue and separation of the acini. In the Cafeteria and Cafeteria + STZ groups, the islets remain identifiable, with less pronounced changes. *Kidney (middle row):* cortical architecture is preserved in the CONTROL group; the STZ and Cafeteria groups display interstitial and periglomerular mononuclear inflammatory infiltrate (arrows), accompanied by thickening of the intertubular matrix and tubular dilation. The Cafeteria + STZ group shows a glomerulus with increased cellularity and retraction of the urinary space. *Liver (lower row):* regular hepatocyte cords in the CONTROL group; the STZ group shows mild to moderate macro- and microvesicular steatosis with diffuse distribution (arrows); the Cafeteria and Cafeteria + STZ groups show foci of portal and lobular mononuclear inflammatory infiltrate (arrows), with focal disorganization of the hepatocyte cords. Scale bars = 100 μm in all images.

Renal histopathological analysis showed preserved overall tissue architecture in both the Healthy and Cafeteria groups. Although mild inflammatory infiltration and tubular alterations were observed in some animals receiving the cafeteria diet, the overall histopathological score was not significantly different from that of the Healthy group (*P* > 0.05). Glomerulosclerosis, arteriolar hyalinosis, and interstitial fibrosis were not observed (Figure 4).

Pancreatic sections showed preserved islet architecture in the Healthy group, whereas some animals in the Cafeteria group exhibited focal peri-islet inflammatory changes and varying degrees of islet alteration. However, the overall histopathological score did not differ significantly between the groups (*P* > 0.05), indicating that the observed pancreatic morphological changes were not statistically significant (Figure 4).

In contrast, visceral adipose tissue showed significant morphological alterations associated with cafeteria diet consumption. Adipocyte hypertrophy differed significantly among the experimental groups (*P* < 0.001). Dunn’s post hoc analysis demonstrated significantly greater adipocyte hypertrophy in the Cafeteria group compared with the Healthy group (*P* < 0.01) and in the Cafeteria + STZ group compared with the Healthy group (*P* < 0.001). Histologically, both cafeteria-fed groups exhibited enlarged and heterogeneous adipocytes, accompanied by fibrous thickening of the interlobular septa and perivascular stroma with focal mononuclear cell infiltration (Figure 5). However, no significant differences were observed for crown-like structures, extracellular matrix fibrosis, or leukocyte infiltration in the evaluated pairwise comparisons. These findings indicate that the cafeteria diet was primarily associated with adipose tissue remodeling and marked adipocyte hypertrophy, whereas the histopathological changes observed in the liver, kidneys, and pancreas did not reach statistical significance.

**Figure 5:**
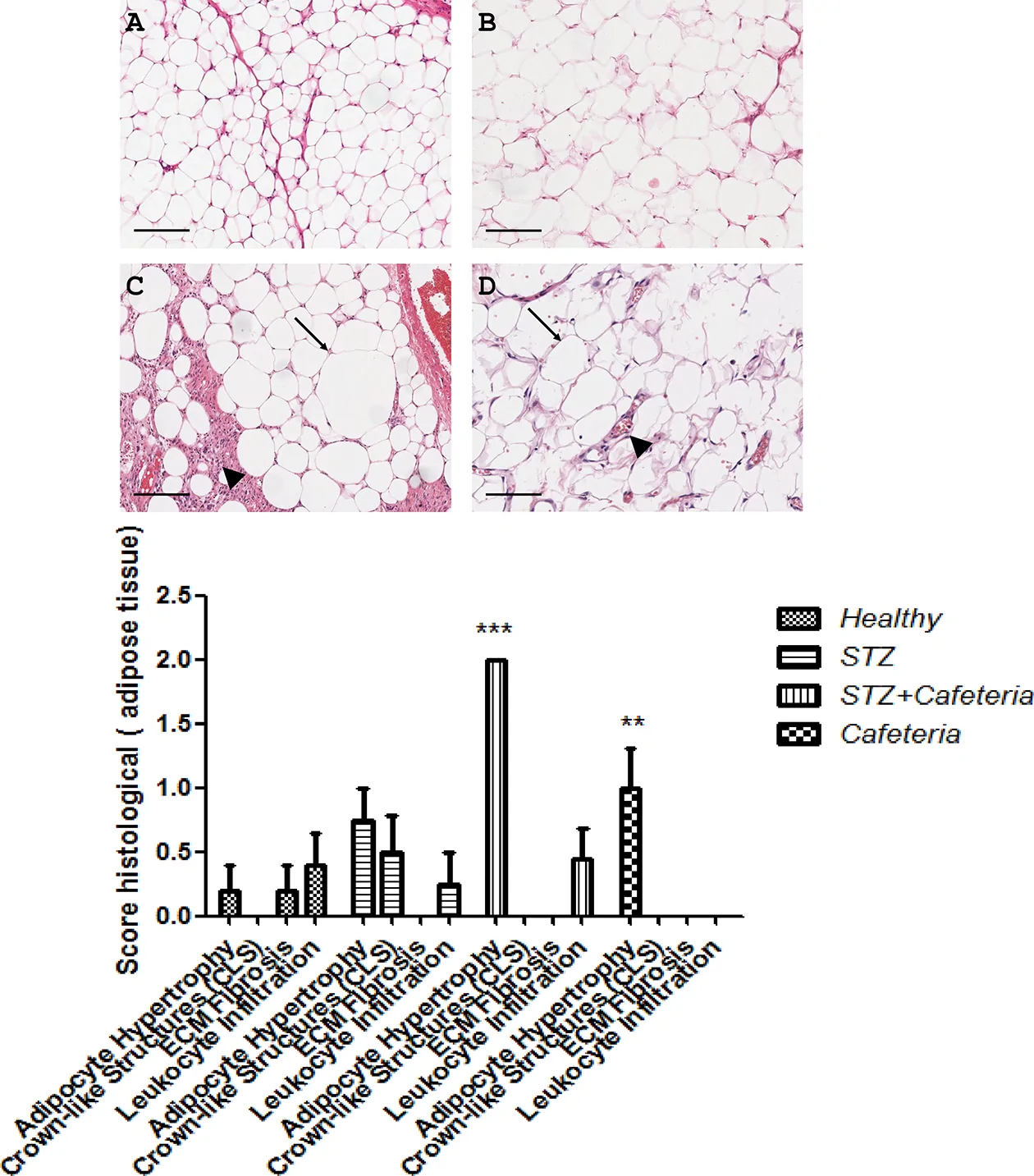
Adipocyte hypertrophy and adipose tissue remodeling in rats with STZ-induced diabetes and/or fed a cafeteria diet Adipose tissue sections stained with H&E. (**A**) Control group, showing adipocytes of uniform size and thin connective tissue septa. (**B**) STZ group, with architecture similar to control and only slight variation in adipocyte diameter. (**C**) Cafeteria group and (**D**) cafeteria + STZ group, both showing marked adipocyte hypertrophy with large, distended cells (arrows), pronounced heterogeneity in cell size, and fibrous thickening of the interlobular septa and perivascular stroma with mononuclear cell infiltration (arrowheads). Arrows = hypertrophic adipocytes; arrowheads = fibrous thickening of the stroma/septa. Scale bars = 100 μm in all images. The results showed significant differences among groups for adipocyte hypertrophy (*P* < 0.001). Dunn’s post hoc analysis showed significantly greater adipocyte hypertrophy in the STZ + Cafeteria group compared with the Healthy group (*P* < 0.001) and in the Cafeteria group compared with the Healthy group (*P* < 0.01). No significant differences were observed for crown-like structures, extracellular matrix fibrosis, or leukocyte infiltration in the indicated pairwise comparisons. **P < 0.01; ***P < 0.001.

## Discussion

The findings of the present study largely support the proposed hypothesis that chronic exposure to a standardized thermally processed cafeteria diet would reproducibly induce an early metabolic syndrome-like phenotype in Wistar rats, characterized by obesity, insulin resistance, low-grade inflammation, adipose remodeling, altered insulin-related gene expression, and gut microbiota disruption. The standardized and processed cafeteria diet used in this experimental protocol reproducibly induced key metabolic features consistent with early metabolic syndrome, including marked increases in body weight, abdominal circumference, Lee index, hyperglycemia, hypertriglyceridemia, increased HOMA-IR, inflammatory activation, adipocyte hypertrophy, down-regulation of muscle *Slc2a4/GLUT4*, adipose leptin up-regulation, and microbial ecological simplification. Thus, the proposed hypothesis was largely supported and strongly corroborated rather than refuted, although some expected late-stage manifestations of metabolic disease—such as overt diabetes, severe adipose inflammation, or advanced organ remodeling—were not consistently observed, reinforcing that this model reproduces an early stage of metabolic syndrome.

Animals exposed to the cafeteria diet showed marked increases in body weight, abdominal circumference, and Lee index compared with animals fed the standard diet. In particular, the Cafeteria group exhibited a body weight gain of 172.3% and abdominal circumference increase of 38.7%, characterizing an obese phenotype, while the Cafeteria + STZ group developed an overweight phenotype with significant metabolic alterations. These findings are consistent with previous studies demonstrating that cafeteria-style diets promote rapid weight gain, adiposity expansion, and metabolic disturbances in rodents [8,9].

These findings reinforce the strong obesogenic potential of hypercaloric diets rich in processed foods, saturated fats, and refined carbohydrates, which mimic dietary patterns commonly associated with the development of metabolic syndrome in humans [6,10]. Cafeteria-type diets can be used to induce more pronounced diabetic phenotypes, particularly when dietary exposure is prolonged or when the diet is combined with pharmacological interventions such as STZ. In particular, cafeteria diet + STZ models have been reported to produce sustained hyperglycemia and other features compatible with experimental diabetes [8,22–24]. However, the phenotype obtained in the present study differs from these models, as the thermally processed cafeteria diet predominantly induced obesity and early metabolic dysfunction rather than a uniformly overt diabetic phenotype [8,22].

Excessive adipose tissue expansion is known to promote adipocyte hypertrophy, increased lipolysis, and ectopic lipid deposition, which are key drivers of metabolic dysfunction. This is corroborated by the histopathological findings of significant adipocyte hypertrophy (*P* < 0.01), despite the absence of crown-like structures, fibrosis, or marked inflammatory infiltration, indicating that adipose tissue remodeling occurs early and precedes overt inflammatory infiltration.

In addition to obesity-related parameters, animals exposed to the cafeteria diet also exhibited biochemical alterations consistent with metabolic dysfunction. Increased fasting glucose levels were observed in both the Cafeteria and Cafeteria + STZ groups. Importantly, the markedly elevated HOMA-IR values observed in cafeteria-fed animals further reinforce the presence of obesity-associated insulin resistance accompanied by compensatory hyperinsulinemia, supporting the interpretation that this model reproduces early metabolic dysfunction compatible with metabolic syndrome rather than overt diabetes. Similar alterations in glucose regulation have been widely reported in rodent models fed cafeteria or Western-style diets, which promote insulin resistance and metabolic imbalance [9,25]. Although glucose measurements may have been partially influenced by the hyperglycemic effect of xylazine anesthesia, the relative differences between groups strongly indicate a diet-associated metabolic disturbance. Xylazine-induced hyperglycemia has been previously described as a result of α2-adrenergic inhibition of insulin secretion [7].

Interestingly, HbA1c levels remained within the normal range for most animals, although a small subset of individuals in the cafeteria diet groups exhibited values compatible with severe diabetes. These findings indicate that the experimental model predominantly reproduces metabolic alterations characteristic of metabolic syndrome rather than overt diabetes. Similar observations have been reported in diet-induced obesity models, where metabolic syndrome features such as obesity, dyslipidemia, and insulin resistance precede the development of chronic hyperglycemia and sustained elevations in HbA1c [3]. Importantly, this molecular impairment occurs in parallel with early pancreatic histopathological alterations, reinforcing the link between insulin resistance and structural endocrine dysfunction. Another important finding was the significant reduction in GLUT4 mRNA expression. The reduced expression of GLUT4, alongside increased fasting glucose, suggests that the cafeteria diet promotes molecular alterations in glucose transport pathways. The synergy between the high lipid fraction and the pro-inflammatory Maillard reaction products likely exacerbated the down-regulation of GLUT4, compromising glucose uptake in insulin-sensitive tissues. Nevertheless, prolonged exposure to hypercaloric diets or increased metabolic susceptibility may eventually lead to more advanced glycemic dysregulation and diabetes-like phenotypes [26].

Chronic low-grade inflammation is a hallmark of metabolic syndrome and plays a critical role in the development of insulin resistance [27]. In the present study, animals exposed to the experimental models exhibited significantly increased levels of pro-inflammatory cytokines IL-1β and IL-6. The high-temperature processing (180°C) of a mixture rich in proteins and reducing sugars (from condensed milk and biscuits) favors the Maillard reaction. Elevated circulating levels of these cytokines have been widely reported in both experimental and clinical studies of obesity and metabolic syndrome, where they contribute to systemic metabolic dysfunction and impaired insulin signaling [28].

Consistent with the inflammatory profile observed systemically, histopathological analysis of pancreatic islets demonstrated significant structural and inflammatory alterations in cafeteria-fed animals. While control animals exhibited preserved islet architecture, cafeteria-fed animals showed increased variability, including peri-insulitis, moderate insulitis, and islet atrophy. These findings, together with the statistical significance observed, indicate that pancreatic tissue is particularly susceptible to early inflammatory and degenerative changes in this model, potentially contributing to altered insulin homeostasis and impaired glucose regulation.

The elevation of these cytokines in cafeteria-fed animals supports the concept that hypercaloric diets promote systemic inflammatory activation. Experimental models using high-fat or Western-style diets consistently demonstrate activation of inflammatory pathways in adipose tissue and peripheral organs [29]. Adipose tissue expansion is known to trigger the release of inflammatory mediators and recruitment of immune cells, particularly macrophages, leading to a state of chronic metabolic inflammation [30].

In adipose tissue, histopathological findings revealed mild adipocyte hypertrophy without significant inflammatory infiltration, fibrosis, or crown-like structures. This suggests that, although adipose expansion was evident, tissue remodeling and inflammatory infiltration had not yet progressed to more advanced stages typically associated with chronic obesity.

Consistent with this mechanism, leptin gene expression was significantly up-regulated in animals receiving the cafeteria diet. Leptin is produced by adipocytes and its expression increases proportionally with adipose tissue mass [31]. Elevated leptin levels are commonly associated with obesity and may reflect the development of leptin resistance, a condition frequently observed in metabolic syndrome and characterized by impaired hypothalamic regulation of energy balance and metabolism [32].

Another important finding of the present study was the significant reduction in GLUT4 mRNA expression observed in animals exposed to the cafeteria diet and the Cafeteria + STZ model. GLUT4 is the main insulin-responsive glucose transporter in skeletal muscle and adipose tissue, and its down-regulation is strongly associated with impaired glucose uptake and insulin resistance [33].

The reduced expression of GLUT4 observed in the present study suggests that the cafeteria diet promotes molecular alterations in glucose transport pathways that may contribute to the metabolic disturbances observed. Experimental and clinical studies have shown that diets rich in saturated fats and refined carbohydrates can impair insulin signaling cascades, reduce GLUT4 expression and translocation, and ultimately compromise glucose uptake in insulin-sensitive tissues [26].

Together, these molecular alterations reinforce the ability of the standardized cafeteria diet to reproduce key pathophysiological mechanisms involved in metabolic syndrome. Alterations in insulin signaling pathways, including decreased GLUT4 expression and impaired glucose transport, are widely recognized as central events in the development of insulin resistance and metabolic syndrome [34].

The gut microbiota analysis further supports the metabolic impact of the cafeteria diet model. Healthy animals exhibited a microbiota profile dominated by *Lactobacillus* and other beneficial bacteria associated with intestinal homeostasis and short-chain fatty acid production [35].

In our study, the Cafeteria group showed a marked predominance of Firmicutes (99.83%), largely represented by *Ligilactobacillus* (99.36 ± 0.61%), indicating a highly selective microbial profile. In contrast, Ramírez-Maldonado et al. reported a more dynamic response to cafeteria feeding in mice, with increased Clostridia and Proteobacteria during the early phase of diet exposure, accompanied by reduced Verrucomicrobiota, particularly *Akkermansia*. At a later time point, Bacteroides and Actinobacteria became more prominent, whereas Lactobacilli were reduced [36]. These differences may reflect variation in animal species, diet composition, duration of exposure, and experimental conditions. Nevertheless, both studies indicate that cafeteria-type diets can markedly alter gut microbial community structure during metabolic dysfunction. In the present study, the absence of a substantial expansion of Enterobacteriaceae or other opportunistic taxa suggests that the microbial alterations observed in the Cafeteria group did not correspond to a generalized enrichment of potentially pathogenic bacteria. Therefore, the pronounced predominance of *Ligilactobacillus* should be interpreted primarily as a taxonomic shift associated with the experimental diet rather than as evidence of a specific pathogenic or protective mechanism. Overall, these findings reinforce the importance of considering the specific composition and duration of cafeteria diets when interpreting microbiota responses in experimental models of metabolic dysfunction.

Taken together, the findings of the present study indicate that the standardized and thermally processed cafeteria diet developed for this experimental protocol successfully reproduced multiple features of metabolic syndrome, including obesity, low-grade systemic inflammation, altered glucose metabolism, insulin resistance, and gut microbiota dysbiosis. Similar metabolic alterations have been described in experimental models using hypercaloric or Western-style diets, reinforcing the translational relevance of diet-induced metabolic models. Taken together, the findings of the present study indicate that the standardized and thermally processed cafeteria diet developed for this experimental protocol successfully reproduced multiple features of metabolic syndrome, including obesity, low-grade systemic inflammation, altered glucose metabolism, insulin resistance, and gut microbiota dysbiosis. Similar metabolic alterations have been described in experimental models using hypercaloric or Western-style diets, reinforcing the translational relevance of diet-induced metabolic models [8,37,38].

Regarding hepatic steatosis, the absence of macrovesicular accumulation—despite marked hyperglycemia and hypertriglyceridemia—suggests that our 102-day protocol captures the incipient stages of metabolic syndrome. At this juncture, while metabolic signaling is severely impaired (as evidenced by the down-regulation of GLUT4), structural hepatic damage has not yet reached a point of no return. This biochemical-histological dissociation underscores the model’s utility in investigating early molecular triggers prior to overt organ failure.

## Conclusion

In conclusion, the standardized and thermally processed cafeteria diet used in the present study induced a multidimensional metabolic phenotype in Wistar rats, characterized by increased body weight and adiposity, low-grade systemic inflammation, alterations in glucose homeostasis and insulin resistance, changes in pancreatic morphology, reduced *Slc2a4* (GLUT4) mRNA expression in skeletal muscle, altered *Leptin* mRNA expression in adipose tissue, and changes in gut microbiota composition. These findings support the ability of the dietary protocol to reproduce relevant features of obesity-associated metabolic dysfunction and metabolic syndrome under the experimental conditions evaluated.

The use of a standardized and thermally processed formulation provides a reproducible experimental approach for investigating diet-associated metabolic alterations. However, the present study does not establish that thermal processing itself is responsible for the observed biological effects, nor does it directly demonstrate mechanisms involving food intake, reward-related feeding behavior, or leptin resistance. Therefore, these mechanisms should be addressed in future comparative studies.

Overall, the present model provides a useful experimental platform for studying obesity-associated metabolic dysfunction and early features of metabolic syndrome, as well as for evaluating potential therapeutic interventions.

## Supplementary Material

Supplementary Figure S1 and Tables S1-S4

## Data Availability

All data supporting the findings of the present study are included within the main manuscript and its supplementary materials. The datasets generated and analyzed during the current study are available in the figures, tables, and supplementary files provided with the article. No additional external datasets were generated or deposited in public repositories. Further information may be made available from the corresponding author upon reasonable request.

## Abbreviations

ALT: alanine aminotransferase
AST: aspartate aminotransferase
ELISA: enzyme-linked immunosorbent assay
GAPDH: glyceraldehyde-3-phosphate dehydrogenase
H&E: hematoxylin and eosin
HbA1c: glycated hemoglobin
HOMA: Homeostatic Model Assessment
STZ: streptozotocin
