# Supplementary material for: A thermally processed cafeteria diet reproducibly mimics early metabolic syndrome through obesity, insulin resistance, and inflammatory activation in Wistar rats
Source: Biosci Rep. 2026 Oct 23;46(11):BSR20260388. doi: 10.1042/BSR20260388 (PMC13694264; doi:10.1042/BSR20260388)
Supplement: Supplementary file 1 [file BSR-2026-0388_supp.pdf]

Suplemmentary materials

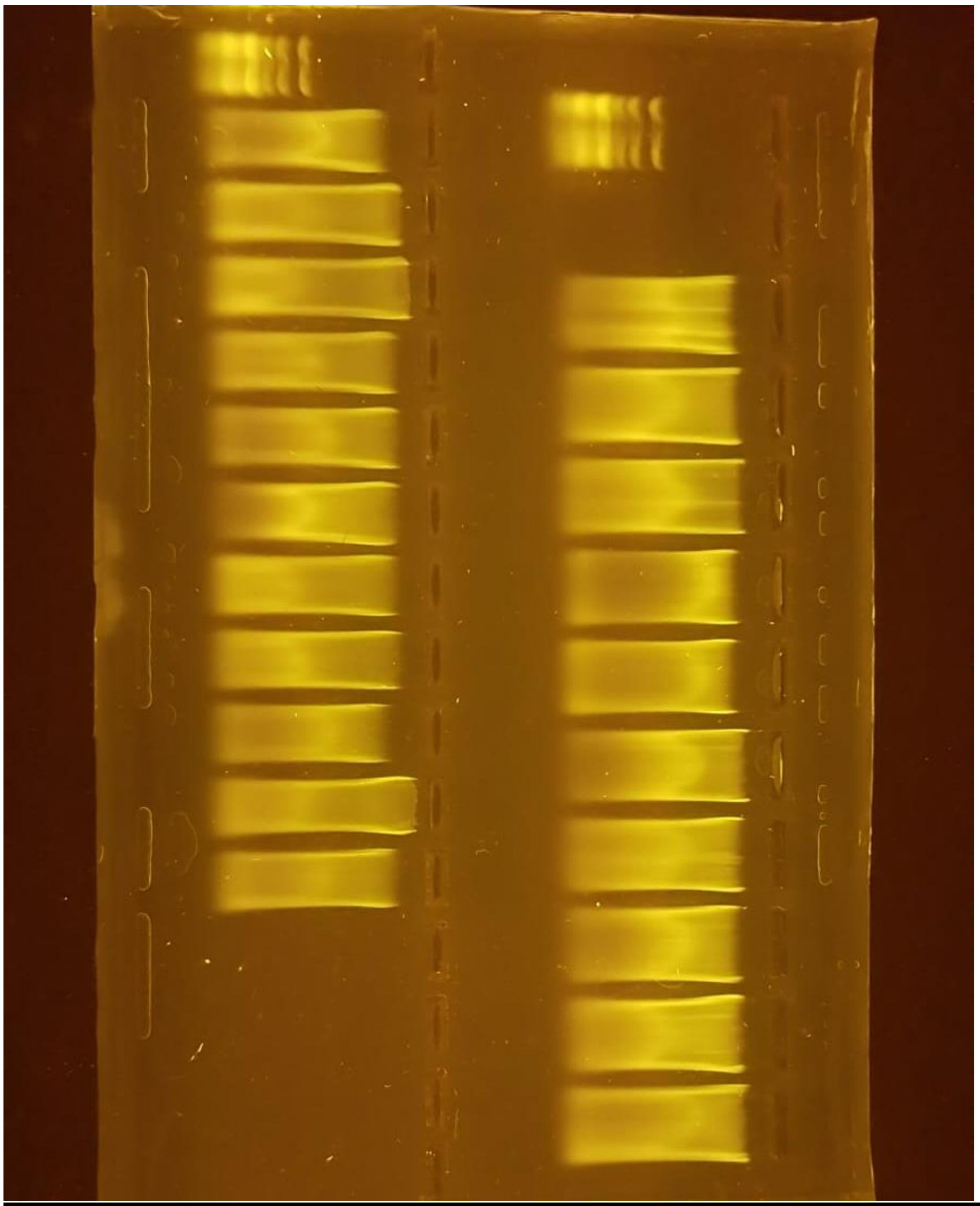

Supplementary Figure S1. Full uncropped 0.8% agarose gel electrophoresis image used for genomic DNA quality control. Electrophoresis was performed in 1× TAE buffer at 110 V for 1 h and visualized using SYBR Safe DNA Gel Stain. GeneRuler 100 bp Plus DNA Ladder (Thermo Scientific) was used as the molecular size marker. This uncropped image is provided in accordance with the journal's data transparency requirements and corresponds to the DNA quality assessment step performed prior to library preparation and Oxford Nanopore sequencing.

Table 1: Individual serum biochemical parameters in male Wistar rats subjected to cafeteria diet and/or streptozotocin exposure during experimental metabolic syndrome induction

SAMPLE ID	GROUP	FRUCTOSAMINE 40 – 140 (µmol/L)	TG 23 – 85 (mg/dL)	CHOL 34 – 70 (mg/dL	HDL-C 14 – 31 (mg/dL)	UREA 34 – 72 (mg/dL)	CREA 0.30 – 0.60 (mg/dL)	UA 0.9 – 1.5 (mg/dL)	TP 5.4 – 7.2 (g/dL)	AST 37 – 196 (U/L)	ALT 29 – 78 (U/L)	AST/ALT ratio 0.5 – 6.8 (U/L)	ALP 88 – 164 (U/L)	LDH 380 - 1587(U/L)	CK-MB 5.9 – 86.6 (U/L)
**A7**	Healthy	138	199	67	66.4	53	0.61	0.7	5.3	133	96	1.4	177	320	404
**A8**	Healthy	153	122	51	51	39	0.80	6.5	6.8	90	59	1.5	173	431	107
**A9**	Healthy	145	123	62	61	36	0.70	3.6	6.4	76	48	1.6	197	342	212
**A10**	Healthy	127	141	65	59.7	35	0.60	0.9	5.9	100	57	1.8	167	491	248
**A11**	Healthy	144	81	47	36.7	35	0.66	5.0	6.2	84	62	1.4	134	297	108
**A12**	Healthy	141	133	64	88.0	43	1.00	1.3	6.1	146	134	1.1	156	496	514
**A13**	STZ	157	148	59	58.0	42	1.10	10.8	5.2	108	66	1.6	136	279	88
**A14**	STZ	164	195	78	82.0	36	0.70	3.3	5.6	169	68	2.5	167	1009	406
**A28**	STZ	111	49	58	-	62	0.46	4.2	5.4	89	37	2.4	105	445	324
**A29**	STZ	98	76	50	-	111	0.59	1.9	7.5	115	36	3.2	124	1101	325
**A30**	STZ	166	141	60	-	72	0.69	1.6	6.3	113	52	2.2	133	1638	1320
**A15**	CAFETERIA + STZ	212	112	53	AI	31	1.10	2.7	5.7	124	33	3.8	370	1252	-
**A16**	CAFETERIA + STZ	211	137	47	AI	32	1.10	12.5	6.3	89	37	2.4	AI	356	-
**A17**	CAFETERIA + STZ	365	280	73	55.0	38	0.70	2.9	6.2	240	73	3.3	169	1509	402
**A19**	CAFETERIA + STZ	103	193	62	68.0	28	0.52	0.7	5.8	73	30	2.4	84	375	274
**A20**	CAFETERIA + STZ	101	121	63	46.0	32	0.70	0.7	6.9	108	66	1.6	275	890	550
**A21**	CAFETERIA + STZ	107	158	57	55.0	33	0.70	2.1	5.9	117	35	3.3	192	945	515
**A22**	CAFETERIA + STZ	124	167	54	61.0	39	0.66	2.4	5.6	98	62	1.6	297	428	320
**A23**	CAFETERIA + STZ	122	124	60	65.0	28	0.67	0.7	6.1	289	63	4.6	207	128	371
**A31**	CAFETERIA + STZ	107	174	60	-	35	0.64	1.6	6.0	87	28	3.1	213	779	633
**A32**	CAFETERIA + STZ	122	45	60	-	37	0.52	2.1	5.7	80	18	4.5	164	1445	629
**A33**	CAFETERIA + STZ	120	137	55	-	34	0.58	3.2	7.0	57	24	2.4	155	481	265
**A34**	CAFETERIA + STZ	-	35	42	-	-	-	1.8	8.6	65	19	3.4	139	774	415
**A35**	CAFETERIA + STZ	108	90	81	-	127	0.63	2.2	6.4	130	27	4.9	-	728	-
**A36**	CAFETERIA + STZ	-	-	-	-	-	-	-	5.5	100	27	3.7	170	739	298

**A24**	CAFETERIA	120	165	53	52.0	31	0.80	2.9	6.9	117	51	2.3	250	762	456
**A25**	CAFETERIA	99	154	54	49.0	24	0.60	0.5	6.3	99	36	2.8	139	445	273
**A26**	CAFETERIA	98	139	55	56.0	32	0.52	0.8	5.8	79	52	1.5	215	478	342
**A27**	CAFETERIA	100	96	45	-	58	0.68	1.5	6.0	78	42	1.9	190	214	144
**A37**	CAFETERIA	194	106	73	-	78	0.60	2.2	5.6	70	72	1.0	534	928	638
**A38**	CAFETERIA	112	111	63	-	68	0.70	1.8	5.0	77	82	0.9	288	676	309
**A39**	CAFETERIA	190	97	70	-	83	0.44	1.1	5.9	78	65	1.2	360	807	534

Table 2: Individual glycated hemoglobin (HbA1c) values and comparative glycemic classification in male Wistar rats subjected to cafeteria diet and/or streptozotocin exposure

Sample	Grupo	HbA1c (%)	Classificação
A7	Health	4,3	Within physiological range
A8	Health	4,3	Within physiological range
A9	Health	4,1	Within physiological range
A10	Health	4,1	Within physiological range
A11	Health	4,2	Within physiological range
A12	Health	4,3	Within physiological range
A13	STZ	4,1	Within physiological range
A14	STZ	4,5	Within physiological range
A28	STZ	4,2	Within physiological range
A29	STZ	4,2	Within physiological range
A30	STZ	4,1	Within physiological range
A15	Cafeteria+STZ	4,5	Within physiological range
A16	Cafeteria+STZ	4,2	Within physiological range
A17	Cafeteria+STZ	4,4	Within physiological range
A18	Cafeteria+STZ	4,4	Within physiological range
A19	Cafeteria+STZ	4,6	Within physiological range
A20	Cafeteria+STZ	4,0	Within physiological range
A21	Cafeteria+STZ	4,2	Within physiological range
A22	Cafeteria+STZ	3,9	Within physiological range
A23	Cafeteria+STZ	3,9	Within physiological range
A31	Cafeteria+STZ	8,1	Markedly elevated relative to healthy controls
A32	Cafeteria+STZ	4,2	Within physiological range
A33	Cafeteria+STZ	4,2	Within physiological range
A34	Cafeteria+STZ	4,5	Within physiological range
A35	Cafeteria+STZ	4,6	Within physiological range
A24	Cafeteria	4,0	Within physiological range
A25	Cafeteria	4,0	Within physiological range
A26	Cafeteria	3,9	Within physiological range
A37	Cafeteria	9,5	Markedly elevated relative to healthy controls
A38	Cafeteria	4,3	Within physiological range
A39	Cafeteria	8,7	Markedly elevated relative to healthy controls

Table 3: Individual hematological parameters in male Wistar rats subjected to cafeteria diet and/or streptozotocin exposure during experimental metabolic syndrome induction

**Sample ID**	**Group**	**RDW-CV** 10 - 14 (%)	**LYM** 0 (%)	**BAND** 0 - 2 (%)	**META** 0 (%)	**MYELO** 0 (%)	**EOS** 0 - 4 (%)	**BASO** 0 - 1 (%)	**PDW** 4.5 - 7.5 (%)	**PCT** 0.20 - 0.99 (%)
**A7**	Healthy	10.1	6	0	0	0	0	0	5.9	0.4
**A8**	Healthy	10.70	5	0	0	0	0	0	6.5	0.43
**A9**	Healthy	10.1	12	0	0	0	0	0	7.8	0.01
**A10**	Healthy	10.1	6	0	0	0	0	0	5.7	0.41
**A11**	Healthy	9.6	6	0	0	0	0	0	6.5	0.32
**A12**	Healthy	10.1	3	1	0	0	0	0	6.5	0.55
**A13**	STZ	10.5	8	1	0	0	0	0	6.5	0.45
**A14**	STZ	9.8	2	0	0	0	0	0	5.9	0.50
**A27**	STZ	10.1	3	0	0	0	0	0	5.9	0.41
**A28**	STZ	10.8	7	0	0	0	0	0	5.7	0.41
**A29**	STZ	10.0	5	0	0	0	0	0	5.9	0.53
**A15**	Cafeteria+STZ	11.9	6	0	0	0	0	0	8.4	0.54
**A16**	Cafeteria+STZ	11.7	7	1	0	0	0	0	5.9	0.37
**A17**	Cafeteria+STZ	10.7	3	1	0	0	0	0	7.3	0.56
**A18**	Cafeteria+STZ	11.6	4	0	0	0	0	0	10	0.03
**A19**	Cafeteria+STZ	11.7	8	0	0	0	0	0	7.8	0.44
**A20**	Cafeteria+STZ	11.3	5	0	0	0	0	0	5.7	0.27
**A21**	Cafeteria+STZ	10.5	5	0	0	0	0	0	5.9	0.39
**A22**	Cafeteria+STZ	10.6	5	0	0	0	0	0	6.5	0.43
**A30**	Cafeteria+STZ	10.1	3	0	0	0	0	0	5.4	0.42
**A31**	Cafeteria+STZ	11.1	3	0	0	0	0	0	7.0	0.43
**A32**	Cafeteria+STZ	10.7	2	0	0	0	0	0	6.5	0.50
**A33**	Cafeteria+STZ	11.5	2	0	0	0	0	0	6.2	0.54
**A34**	Cafeteria+STZ	10.8	4	1	0	0	0	0	6.5	0.49
**A35**	Cafeteria+STZ	10.8	3	0	0	0	0	0	6.7	0.30
**A23**	cafeteria	11.3	3	1	0	0	0	0	5.9	0.37
**A24**	cafeteria	10.7	5	0	0	0	0	0	5.7	0.4
**A25**	cafeteria	11.3	4	0	0	0	0	0	5.9	0.46
**A26**	cafeteria	11.3	2	0	0	0	0	0	5.4	0.41
**A37**	cafeteria	11.2	2	1	0	0	0	0	7.0	0.41
**A38**	cafeteria	10.9	2	0	0	0	0	0	5.9	0.41
**A39**	cafeteria	11.2	4	0	0	0	0	0	7.5	0.52

Table 4. Fasting glucose, serum insulin levels, HOMA-IR and HOMA-β index across experimental groups

Groups	GLU (mg/dL)	Insulin (pg/mL)	HOMA-IR	HOMA-β
Health	254.83 ± 44.26	5748.97±1339.76	105.88±38.71	319.25±30.65
STZ	257.20 ± 105.83	7478.40±2164.66	122.79±59.72	1941.06 ± 1612.04
Stz+ cafeteria	391.21 ±105.25	7755.21±1882.86	198.67±54.92	319.78 ± 69.73
Cafeteria	452.29 ± 126.66	7061.49±700.47	229.19±73.02	201.71± 18.52

Data are presented as Mean ± SD. Statistical comparisons for HOMA-IR were conducted using One-Way ANOVA ($F = 6.054$, $p = 0.0015$) followed by Tukey's *post-hoc* test for multiple comparisons. Post-hoc analysis for HOMA-IR revealed that: HOMA-IR levels were significantly higher in the Cafeteria group compared to the Health (Control) group ($p = 0.0053$) and the STZ group ($p = 0.0448$). HOMA-IR levels were significantly higher in the STZ + Cafeteria group compared to the Health (Control) group ($p = 0.0268$).
